# Plasma proteome and incident myocardial infarction: sex-specific differences

**DOI:** 10.1093/eurheartj/ehae658

**Published:** 2024-10-14

**Authors:** Olga E Titova, Shuai Yuan, Liisa Byberg, John A Baron, Lars Lind, Karl Michaëlsson, Susanna C Larsson

**Affiliations:** Unit of Medical Epidemiology, Department of Surgical Sciences, Uppsala University, Dag Hammarskjölds väg 14 B, 75185, Uppsala, Sweden; Unit of Cardiovascular and Nutritional Epidemiology, Institute of Environmental Medicine, Karolinska Institutet, Stockholm, Sweden; Unit of Medical Epidemiology, Department of Surgical Sciences, Uppsala University, Dag Hammarskjölds väg 14 B, 75185, Uppsala, Sweden; Unit of Medical Epidemiology, Department of Surgical Sciences, Uppsala University, Dag Hammarskjölds väg 14 B, 75185, Uppsala, Sweden; Department of Medicine, University of North Carolina School of Medicine, Chapel Hill, NC, USA; Department of Epidemiology, Gillings School of Global Public Health, University of North Carolina, Chapel Hill, NC, USA; Unit of Cardiovascular Epidemiology, Department of Medical Sciences, Uppsala University, Uppsala, Sweden; Unit of Medical Epidemiology, Department of Surgical Sciences, Uppsala University, Dag Hammarskjölds väg 14 B, 75185, Uppsala, Sweden; Unit of Medical Epidemiology, Department of Surgical Sciences, Uppsala University, Dag Hammarskjölds väg 14 B, 75185, Uppsala, Sweden; Unit of Cardiovascular and Nutritional Epidemiology, Institute of Environmental Medicine, Karolinska Institutet, Stockholm, Sweden

**Keywords:** Cohort, Proteomics, Myocardial infarction, Mendelian randomization

## Abstract

**Background and aims:**

Few population-based cohort studies, including both men and women, have explored circulating proteins associated with incident myocardial infarction (MI). This study investigated the relationships between circulating cardiometabolic-related proteins and MI risk using cohort-based and Mendelian randomization (MR) analyses and explored potential sex-specific differences.

**Methods:**

The discovery cohort included 11 751 Swedish adults (55–93 years). Data on 259 proteins assessed with Olink proximity extension assays, biochemical, and questionnaire-based information were used. Participants were followed up for incident MI and death over 8 years through linkage to Swedish registers. Replication analyses were conducted on the UK Biobank sample (*n* = 51 613). In MR analyses, index *cis*-genetic variants strongly related to the proteins were used as instrumental variables. Genetic association summary statistic data for MI were obtained from the CARDIoGRAMplusC4D consortium and FinnGen.

**Results:**

Forty-five proteins were associated with incident MI in discovery and replication samples following adjustment for potential confounders and multiple testing. In the secondary analysis, 13 of the protein associations were sex-specific, with most associations identified among women. In MR analysis, genetically predicted higher levels of renin, follistatin, and retinoic acid receptor responder protein 2 were linked to an increased risk of MI. Tissue factor pathway inhibitor, tumor necrosis factor receptors 1 and 2, placenta growth factor had an inverse association with MI.

**Conclusions:**

This study identified both new and confirmed previously established associations between circulating proteins and incident MI and, for the first time, suggested sex-specific patterns in multiple protein-MI associations.

## Structured Graphical Abstract

**Structured Graphical Abstract:**
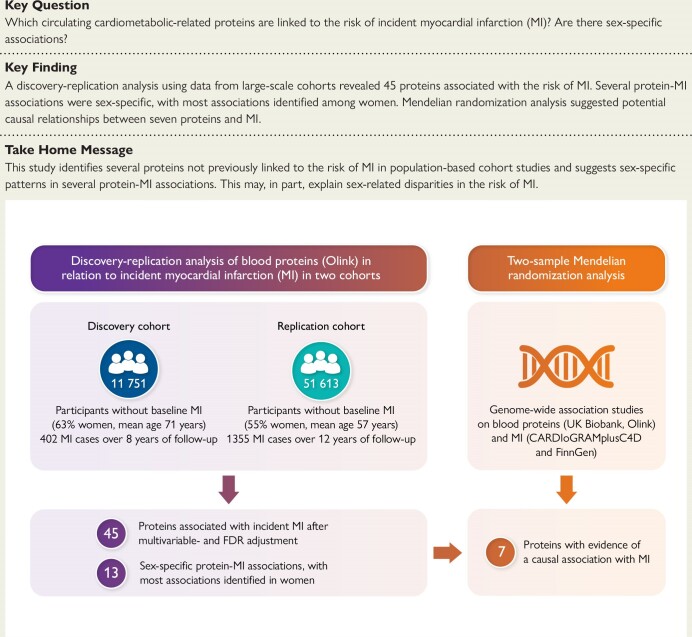
Summary of the main results of the cohort-based and Mendelian randomization analyses on the association between circulating proteins and incident myocardial infarction. FDR, false discovery rate; MI, myocardial infarction.

**See the editorial comment for this article ‘Steps towards curing Yentl syndrome: appraising sex differences in circulating proteins and incident myocardial infarction’, by A. K. Barton *et al.*, https://doi.org/10.1093/eurheartj/ehae657.**

## Introduction

Cardiovascular diseases (CVD), including myocardial infarction (MI), remain the leading cause of premature death and disability worldwide. Atherosclerosis is the most common cause of MI.^1^ Major risk factors for atherosclerosis and MI include diabetes mellitus, dyslipidemia, hypertension, obesity, and smoking.^2^ However, the underlying molecular pathways are poorly understood, and sex differences in the risk of MI have been observed.^3^ Sex-related disparities in the risk of MI may not solely be attributed to lifestyle behaviours (e.g. higher smoking rates in men and differences in dietary patterns) or biological factors (e.g. sex hormones). Still, they could also potentially be explained by differences in proteomic profiles. Whether associations between circulating proteins and the risk of incident MI differ by sex remains unclear.

Discovery and replication of biomarker associations with MI may provide insights into the molecular pathways of disease development, prediction of adverse outcomes, accuracy of diagnosis, and identification of potential therapeutic targets. Most of the current evidence regarding the association between biomarkers and MI is based on prognostic studies focused on specific proteins in patient populations with a history of coronary heart disease (CHD),^4–7^ or only include participants of one sex.^8,9^ Large cohort studies with complete and accurate outcome ascertainment are needed to replicate previous findings, discover novel associations with incident acute MI, consider critical confounding factors, and limit reverse causality, selection bias, and survival bias.

Multiplex proteomic assays offer a simultaneous investigation of a large number of protein biomarkers with potential clinical relevance for disease development. Only a few cohort studies have investigated the association between multiple circulating proteins and the risk of incident MI or composite end-point of MI and stroke.^10–13^ Importantly, potential sex differences in the protein-MI associations have not been explored in cohort studies. In addition, the causality and direction of the associations are often not clear, as observational studies are susceptible to confounding and reverse causality. Mendelian randomization (MR) utilizes genetic variants that are strongly associated with a risk factor as instrumental variables and have the potential to provide less biased evidence for observed associations.^14^

In this study, we investigated the associations between circulating cardiometabolic proteins and the risk of MI in a population-based cohort of 11 751 Swedish women and men. The UK Biobank (*n* = 51 613) served as a replication cohort. In addition, sex-specific associations between circulating proteins and MI risk were explored. We also used the MR design to investigate whether the identified proteins may have a causal role in the development of MI.

## Methods

### Study population

#### SIMPLER (discovery sample)

We used data from the two clinical subcohorts of the National Research Infrastructure SIMPLER (Swedish Infrastructure for Medical Population-based Life-course Environmental Research). Participants in these subcohorts, from two neighboring Swedish counties, were born between 1920 and 1952 and randomly selected from the two larger population-based SIMPLER cohorts, the Swedish Mammography Cohort (SMC) and the Cohort of Swedish Men (COSM). Details of the study cohorts are shown in the Supplementary data online and have been reported elsewhere.^15^ Briefly, in 2003–09, women living in Uppsala County who had previously participated in SMC were randomly chosen for clinical examination and the collection of biosamples (here referred to as the *Uppsala cohort*). Previously, participants had responded to lifestyle, food frequency, and health questionnaires in 1987–90, 1997, 2008–09, and up to one month before the clinical examination. In 2010–19, women and men living in Västmanland County who participated earlier in SMC or COSM were invited to take part in a clinical investigation similar to the one described for the Uppsala cohort (referred to as the *Västmanland cohort*). Information on lifestyle and health characteristics was obtained with structured questionnaires at baseline supplemented with the national registry information.

From the initial sample of 12 314 participants, we excluded individuals diagnosed with MI before baseline according to the International Classification of Diseases, ICD 9th and 10th Revision (*n* = 563), as verified through linkage to the Swedish National Patient and Death Registers. This left 11 751 eligible participants in the final cohort (7423 women and 4328 men) with a mean baseline age of 71 (age range, 55–93) years.

#### UK biobank (replication sample)

The UK Biobank is a multi-center cohort study conducted across the UK (https://www.ukbiobank.ac.uk).^16^ In 2006–10, over 500 000 individuals aged 40–69 underwent physical measurements and blood sample collection and provided questionnaire-based information. The present study used data from 51 613 individuals with valid proteomics data (see Supplementary data online).

### Proteomic analysis

The proteomic analyses in SIMPLER subcohorts have been previously described,^17,18^ and the details are shown in the Supplementary data online. Three high-throughput multiplex immunoassays were used to measure 276 protein biomarkers: the Olink Proseek Multiplex CVD II, CVD III, and Metabolism (SciLifeLab, Uppsala, Sweden), each measuring simultaneously 92 selected proteins related to CVD or metabolism. Protein names and abbreviations can be found in the Supplementary data online, *Table S1*. In previous proteomic analysis based on a subsample of SIMPLER, the *Uppsala cohort,* included in this analysis, no substantial systematic drift between analysis plates was detected.^19^ The results provide relative values and normalized protein expression (NPX) data, which are log2 transformed; one unit higher NPX represents an approximate doubling of the protein concentration. In addition, bridge sample normalization using 100 bridging samples was performed by Olink Bioscience, Uppsala. Seventeen proteins with more than 50% samples below the limit of detection (LOD) were excluded from the analysis by the manufacturer’s recommendations, leaving 259 proteins in our analyses (see Supplementary data online, *Table S2*). Values below the LOD were used as provided by the manufacturer. In the analysis of the CVD II, CVD III, and Metabolism panels, 448 (3.6% of 12 314 analyzed specimens), 97 (0.8%), and 85 (0.7%) samples, respectively, did not pass the manufacturer’s quality control and were therefore set to missing before multiple imputation. SPON1 and GP6 values were available only in the Västmanland cohort; due to a large number of missing values, multiple imputation was not performed, and therefore, the sample size was smaller in these analyses (*n* = 7218). In the UK Biobank, plasma levels of 2923 proteins were successfully measured by the Olink proteomics assay Explore (OLINK, Uppsala, Sweden).^20^

### Assessment of covariates

In SIMPLER, information on diet, health, and lifestyle was obtained through a structured questionnaire the participants completed up to approximately one month before the clinical examination. The dietary intake during the previous year was assessed with a valid and reproducible 96-item food-frequency questionnaire.^17^ A modified Dietary Approaches to Stop Hypertension (mDASH) score was used to determine overall dietary quality.^21^ Participants visited a test center to collect physical measurements and blood samples. Weight (kg), height (cm), levels of fasting glucose, blood pressure, low-density lipoprotein (LDL), and high-density lipoprotein (HDL) cholesterol were measured at the clinical examinations. Details for the UK Biobank are shown in the Supplementary data online.

### Identification of incident MI outcomes and follow-up

In SIMPLER, information regarding MI and death was obtained by linkage to the Swedish National Patient Register and the Cause of Death Register using the unique personal identification number assigned to all Swedish residents. The Patient Register contains all inpatient diagnoses since 1987 and outpatient specialist care since 2001.^22^ We defined incident MI as the first occurrence of the main diagnosis in the Patient Register according to the ICD-10 code I21. Participants were followed up from the baseline (i.e. date of clinical examination) to the date of diagnosis of MI, death from any cause, or 31 December 2020, whichever occurred first. Concerning additional all-cause mortality analysis in SIMPLER, individuals were followed up from the baseline to the date of death from any cause, or 31 December 2020. In the UK Biobank, information on the first MI (ICD-10 code I21) or death was obtained from hospital records and death certificates. Participants were followed up from the date of clinical examination to the date of diagnosis of incident MI, death from any cause, or the end of follow-up, whichever occurred first.

### Genetic data for MR analysis

Proteins discovered and replicated in our cohort study were included in the MR analysis. Summary statistic genetic data on MI were obtained from the genome-wide association meta-analysis for the CARDIoGRAMplusC4D Consortium,^23^ which included 43 676 participants with and 128 199 participants without MI, as well as from the FinnGen R10,^24^ which included 26 060 individuals with and 343 079 without MI.

For the selection of instrumental variables, index *cis*-genetic variants (the SNP in the protein-encoding gene with the smallest *P* value) associated with circulating proteins at the genome-wide significance threshold (*P* < 5 × 10^−8^) were identified from a large genome-wide association study (GWAS), in which protein levels were measured with the Olink technique (n = 54 219).^25^ Thus, one cis-pQTL SNP with the strongest association with each protein was used. Missing SNPs were replaced by proxy SNPs with high linkage disequilibrium *r^2^* ≥ 0.8. All SNPs used had good strength with estimated *F* statistics > 10.^26^ Details of the SNPs used as instrumental variables are presented in Supplementary data online, *Table S3*.

### Statistical analysis

#### Cohort analyses

In the first step, analyses were conducted using a discovery and replication approach, i.e. the SIMPLER sample was used for discovery, and the UK Biobank was used for replication (see Supplementary data online, *Figure S1*). To make results comparable across all 259 proteins, values of circulating proteins were standardized (mean = 0, standard deviation, SD = 1) before the statistical analyses. Details of the replication analysis in the UK Biobank are shown in Supplementary data online.

In the discovery sample (n = 11 751 with 402 incident MI cases), separate multivariable Cox regression analyses were conducted for each of the 259 proteins. The statistical model included age (as the time scale), sex, education (less than high school, high school, or university), project run (Uppsala or Västmanland cohorts), current cigarette smoking (no/yes), alcohol consumption (g/day), walking/cycling (<20 min/day; 20–40 min/day; 40–60 min/day; 60–90 min/day; and >90 min/day), exercise (<1 h/week; 1 h/week; 2–3 h/week; 4–5 h/week; and >5 h/week), adherence to the mDASH diet (dietary score), body mass index (BMI), systolic blood pressure, glucose, LDL and HDL cholesterol. Potential confounders were selected using directed acyclic graphs.^27^ A 5% false discovery rate (FDR) using the Benjamini and Hochberg procedure was applied to account for multiple testing.^28^ Proteins that passed the FDR were included in the replication analysis based on the UK Biobank (n = 51 613 with 1355 incident MI cases) using a regression model adjusted for age, ethnicity (white, black, Asian, and other), Townsend social deprivation index, smoking (never, previous or current), prevalent diabetes (yes/no), systolic blood pressure, BMI, LDL cholesterol, and HDL cholesterol, and fasting time (Supplementary data online). We considered the association of individual proteins and the risk of MI to be replicated if the nominal *P*-value in the replication sample was < 0.05. Correlations between proteins were estimated using Pearson correlation analysis.

In the secondary analyses, sex-specific associations between the 259 circulating proteins and incident MI were assessed using multivariable Cox proportional hazards regression analysis using the same discovery and replication approach (see Supplementary data online, *Figure S1*). First, the multivariable model (same as described for primary analysis) was used on the SIMPLER sample, with the added interaction term protein × sex. Given the limited power of interaction analyses, a FDR adjustment was not applied in this step. Proteins with a nominal *P*-value for interaction term < 0.05 were taken further to validate the interaction term between protein × sex in the UK Biobank. Replicated proteins were included in a sex-specific, fully adjusted analysis of the link between proteins and the risk of MI in discovery and replication samples. The same regression models described for the primary analysis were applied.

Among the 11 751 SIMPLER participants, 22% had missing information on systolic blood pressure, 16% on fasting glucose concentration, 2% on education, 7% on physical activity variables, and 6% on cigarette smoking. Information on adherence to DASH diet, BMI, LDL cholesterol, HDL cholesterol, and alcohol intake was missing in <1%. Missing information on covariates and circulating proteins was imputed using multiple imputations with chained equations with 20 imputation cycles. We conducted additional analyses to explore the association of proteins identified and replicated in the primary analysis with all-cause mortality using SIMPLER cohorts (n = 12 314). All statistical analyses in the cohort analysis were performed using Stata (StataCorp, College Station, TX, USA).

#### Mendelian randomization analysis for identified proteins related to MI

We employed a two-sample MR approach.^14^ The Wald ratio method was used to obtain ratio estimates for each SNP, as the beta coefficient for the SNP-MI association was divided by the beta coefficient for the SNP-protein association. We conducted MR analyses for 38 of the 45 proteins identified and replicated in the primary analysis based on Cox regression models separately for two outcome data sources (CARDIoGRAMplusC4D Consortium and FinnGen) and combined. We did not perform the MR analysis for seven proteins as there was no suitable instrumental variable in protein-related GWAS data, proxy SNPs, or a proxy SNP related to MI in CARDIoGRAMplusC4D or FinnGen (see Supplementary data online, *Table S3*). The fixed-effect model was used for meta-analysis of the results if the data for the same protein was available from two outcome data sources. MR analyses were conducted using the ‘TwoSampleMR’ and ‘MendelianRandomization’ packages in R software (4.4.1).

#### Evaluation of druggability of proteins associated with MI in MR analysis

The DrugBank (https://go.drugbank.com/) and ChEMBL (https://www.ebi.ac.uk/chembl/) databases were used to explore the druggability of proteins related to MI in the primary cohort-based analyses and after MR analyses. Supplementary data online, *Table S12* shows the identified proteins’ drug names, indications, biological function, and drug development details.

## Results

### Cohort-base analyses

Baseline characteristics of SIMPLER participants are presented in *Table 1* and Supplementary data online, *Table S4*. In the cohort of 11 751 participants, 402 had a first diagnosis of MI during a mean follow-up time of 8.3 years. A total of 215 first MIs were diagnosed in women and 187 in men. Information about UK Biobank participants is shown in Supplementary data online, *Table S5*.

**Table 1:** Baseline characteristics of the study participants in the discovery cohort SIMPLER

Characteristics	All	Women	Men
Number of participants	11 751	7423	4328
Age at baseline, years, *mean (SD)*	70.9 (6.8)	69.3 (6.7)	73.6 (6.0)
Education > 12 years, *n (%)*	3340 (29.1)	2392 (33.4)	948 (21.9)
Current cigarette smoking, *n (%)*^a^	3457 (31.4)	1353 (19.6)	2104 (51.2)
Alcohol intake, g/day, *median (Q1-Q3)*	5.0 (1.4–10.8)	3.8 (1.0–8.5)	7.8 (2.8–14.8)
Walking/bicycling >40 min/day, *n (%)*	4206 (38.4)	2599 (38.7)	1607 (37.9)
Exercise ≥ 2 h/week, *n (%)*	4195 (38.4)	3212 (48.0)	983 (23.2)
mDASH Diet score, *mean (SD)*	18.2 (3.7)	18.7 (3.6)	17.3 (3.6)
Systolic blood pressure, mmHg, *means (SD)*	140.4 (17.8)	138.9 (18.5)	142.2 (16.9)
Fasting glucose, mmol/L, *mean (SD)*	5.8 (1.5)	5.6 (1.6)	6.0 (1.3)
LDL-C, mmol/L, *mean (SD)*	3.3 (1.0)	3.5 (1.0)	3.1 (1.0)
HDL-C, mmol/L, *mean (SD)*	1.5 (0.4)	1.6 (0.4)	1.4 (0.3)
Body mass index, kg/m^2^, *mean (SD)*	26.3 (4.1)	26.2 (4.4)	26.5 (3.7)
Incident MI during follow-up, *n*	402	215	187
Age at diagnosis, years, *mean (SD)*	79.5 (7.1)	79.3 (7.3)	79.6 (6.8)

mDASH, modified Dietary Approaches to Stop Hypertension; LDL-C, low-density lipoprotein cholesterol; HDL-C, high-density lipoprotein cholesterol; MI, myocardial infarction; Q1, lower quartile; Q3, upper quartile.

^a^Current smoking included smoking occasionally or regularly.

**Figure 1:**
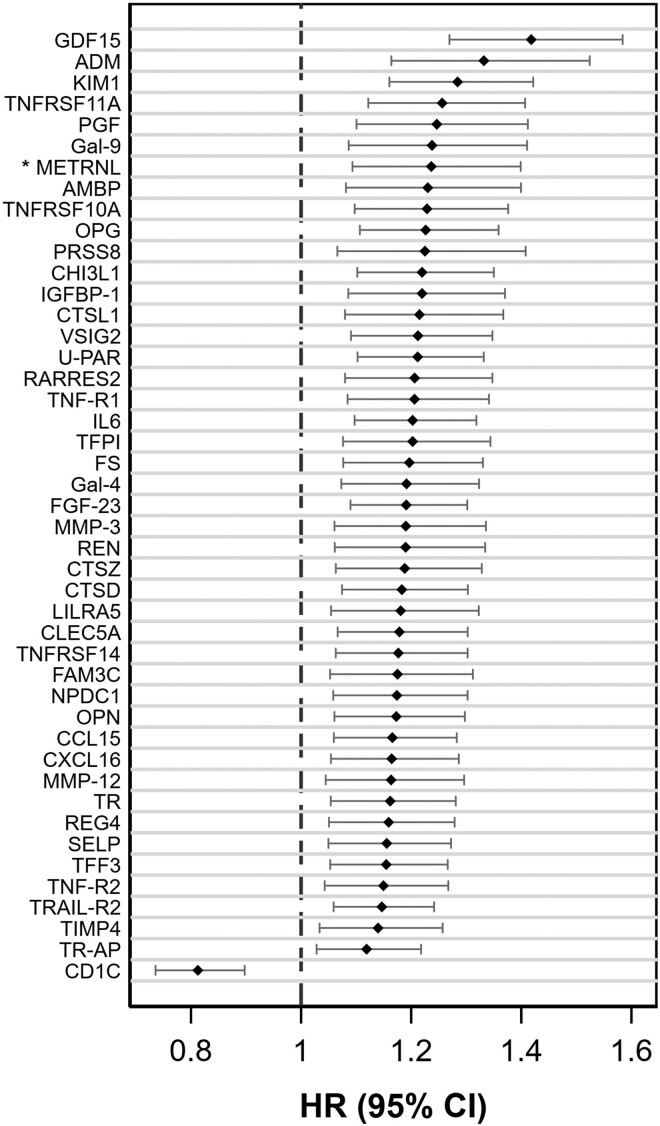
Association of 45 replicated circulating proteins with incident myocardial infarction in SIMPLER cohorts (n = 11 751). Hazard ratios (HR) with 95% confidence intervals (CI) are expressed per standard deviation unit change in protein measurements. Proteins were identified and passed FDR adjustment in the discovery sample, were validated in a replication sample (*P* < 0.05), and had *P* < 0.05 in the multivariable analysis. The models were adjusted for age (as the time scale), sex, education attainment, project, baseline cigarette smoking, alcohol consumption, walking/bicycling, exercise, adherence to the mDASH diet, body mass index, systolic blood pressure, and blood levels of fasting glucose, low-density lipoprotein cholesterol, and high-density lipoprotein cholesterol. * Protein METRNL was not available in the UK Biobank data and, therefore, was not replicated

In the discovery sample (SIMPLER), 51 out of 259 circulating proteins were associated with the risk of incident MI following multiple comparison adjustments in a fully adjusted model. Of these 51 proteins, 44 were associated with MI in the UK Biobank replication sample in a multivariable model (n = 51,613, 1355 MI cases, mean follow-up time of 12.3 years). Although the nominal *P*-value of <0.05 was used to consider a protein to be replicated, an additional multiple comparison adjustment revealed that all 44 proteins passed FDR. The protein METRNL was unavailable in the UK Biobank and, therefore, was not replicated but included in the results based on data from SIMPLER only (*Figure 1* and Supplementary data online, Tables 6 and 7). The majority of the proteins conferred a higher risk of MI, with the strongest association for growth differentiation factor 15 (GDF15; HR per SD 1.42 (95% CI 1.27–1.58) in SIMPLER), followed (in descending order of magnitude) by adrenomedullin (ADM), kidney injury molecule 1 (KIM-1), and tumor necrosis factor receptor superfamily member 11A (TNFRSF11A). In contrast, circulating T-cell surface glycoprotein CD1c (CD1C) levels were associated with a lower risk of MI (HR per SD, 0.81; 95% CI 0.74–0.90, SIMPLER).

Several of the 45 proteins linked to MI were highly correlated. For example, GDF15 was strongly related to tumor necrosis factor receptor 1, TNF-R1 (*r* = 0.73, *P* < 0.001), urokinase plasminogen activator surface receptor, U-PAR (*r* = 0.70, *P* < 0.001), tumor necrosis factor receptor 2, TNF-R2 (*r* = 0.64, *P* < 0.001), neural proliferation differentiation and control protein 1, NPDC1 (*r* = 0.60, *P* < 0.001), protein FAM3C (*r* = 0.60, *P* < 0.001), TNFRSF11A (*r* = 0.60, *P* < 0.001), osteoprotegerin, OPG (r = 0.58, *P* < 0.001), meteorin-like protein, METRNL (r = 0.58, *P* < 0.001), adrenomedullin, ADM (r = 0.56, *P* < 0.001), and retinoic acid receptor responder protein 2, RARRES2 (r = 0.55, *P* < 0.001) (see Supplementary data online, *Figure S2*). Additional multivariable analysis revealed that all 45 proteins linked to MI in primary analysis were associated with all-cause mortality (n of cases = 2147) in SIMPLER (see Supplementary data online, *Table S11*).

**Figure 2:**
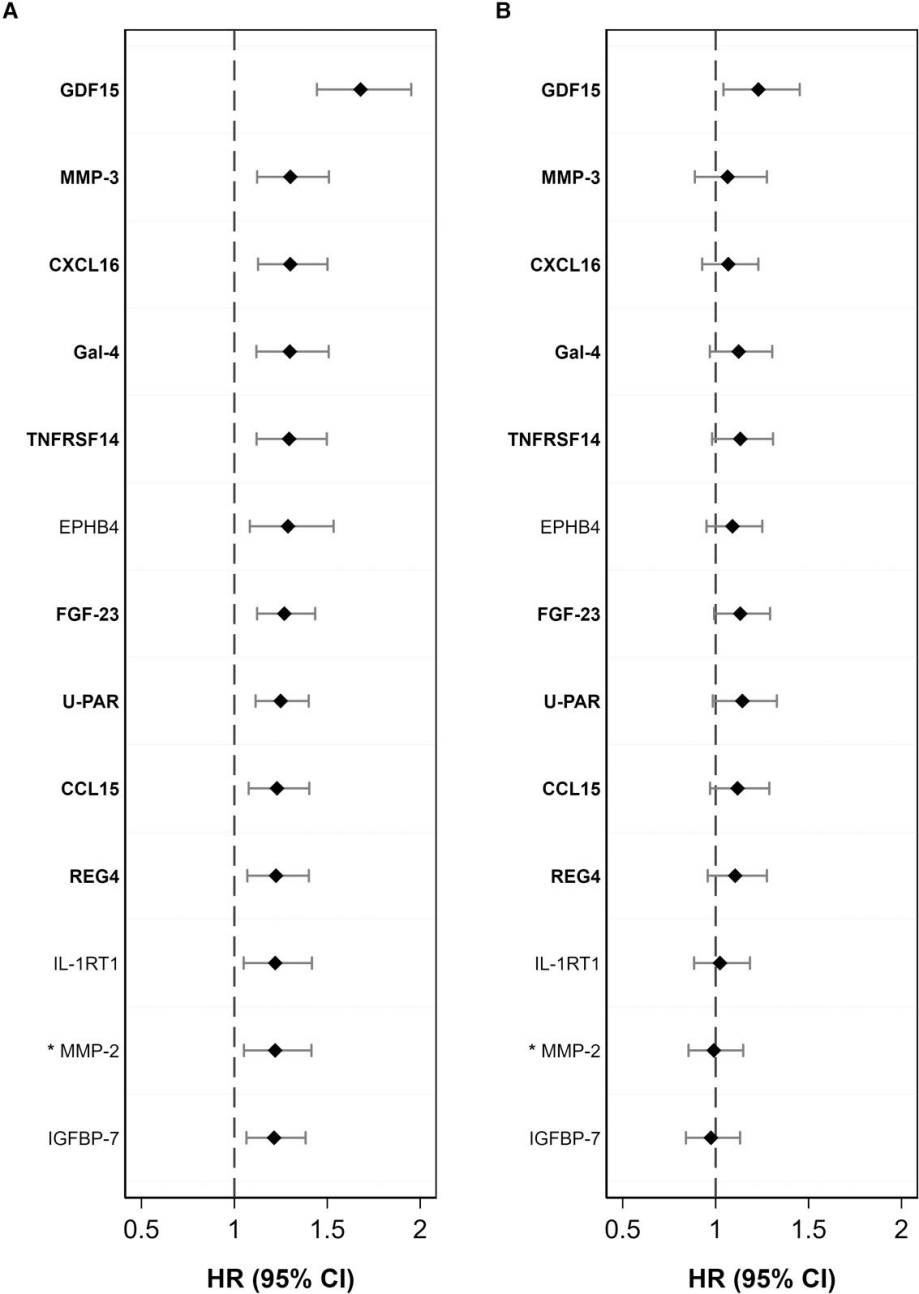
Sex-specific association between replicated circulating proteins and incident myocardial infarction in SIMPLER cohorts. Results for women are shown in the panel A, and for men – panel B. Only proteins that had a statistically significant interaction term of protein × sex in relation to MI in SIMPLER and were replicated in the UK Biobank were included. Hazard ratios (HR) with 95% confidence intervals are expressed per standard deviation unit change in protein measurements. The models were adjusted for age (as the time scale), education attainment, project, baseline cigarette smoking, alcohol consumption, walking/bicycling, exercise, adherence to the mDASH diet, body mass index, systolic blood pressure, and blood levels of fasting glucose, low-density lipoprotein cholesterol, and high-density lipoprotein cholesterol. Proteins identified in the primary analysis based on the entire cohort are highlighted in bold. * Protein MMP-2 was not available in the UK Biobank data and, therefore, was not replicated

In the secondary analysis, 41 statistically significant protein x sex interaction terms were identified in the multivariable analysis in SIMPLER. Of those, 12 were replicated in the multivariable analysis using UK Biobank (*P* < 0.05); one protein, MMP-2, was not available in the UK Biobank data and was therefore not replicated but included in the results based on clear findings in SIMPLER alone. *Figure 2* and Supplementary data online, Tables 8 and 9 show the results of fully adjusted sex-specific analyses.

All these 13 proteins were associated with MI in women in multivariable analyses in the discovery and replication samples (*P* < .05), and all these associations passed FDR adjustment (*Figure 2* and Supplementary data online, *Table S8*). In contrast, in SIMPLER, only GDF15 was linked to the risk of MI in men (*P* < .05). In the UK Biobank, 9 of 12 proteins were related to MI in men (*P* < 0.05); however, the associations were considerably weaker among men compared with women (*Figure 2* and Supplementary data online, *Table S9*).

### MR analysis

**Figure 3:**
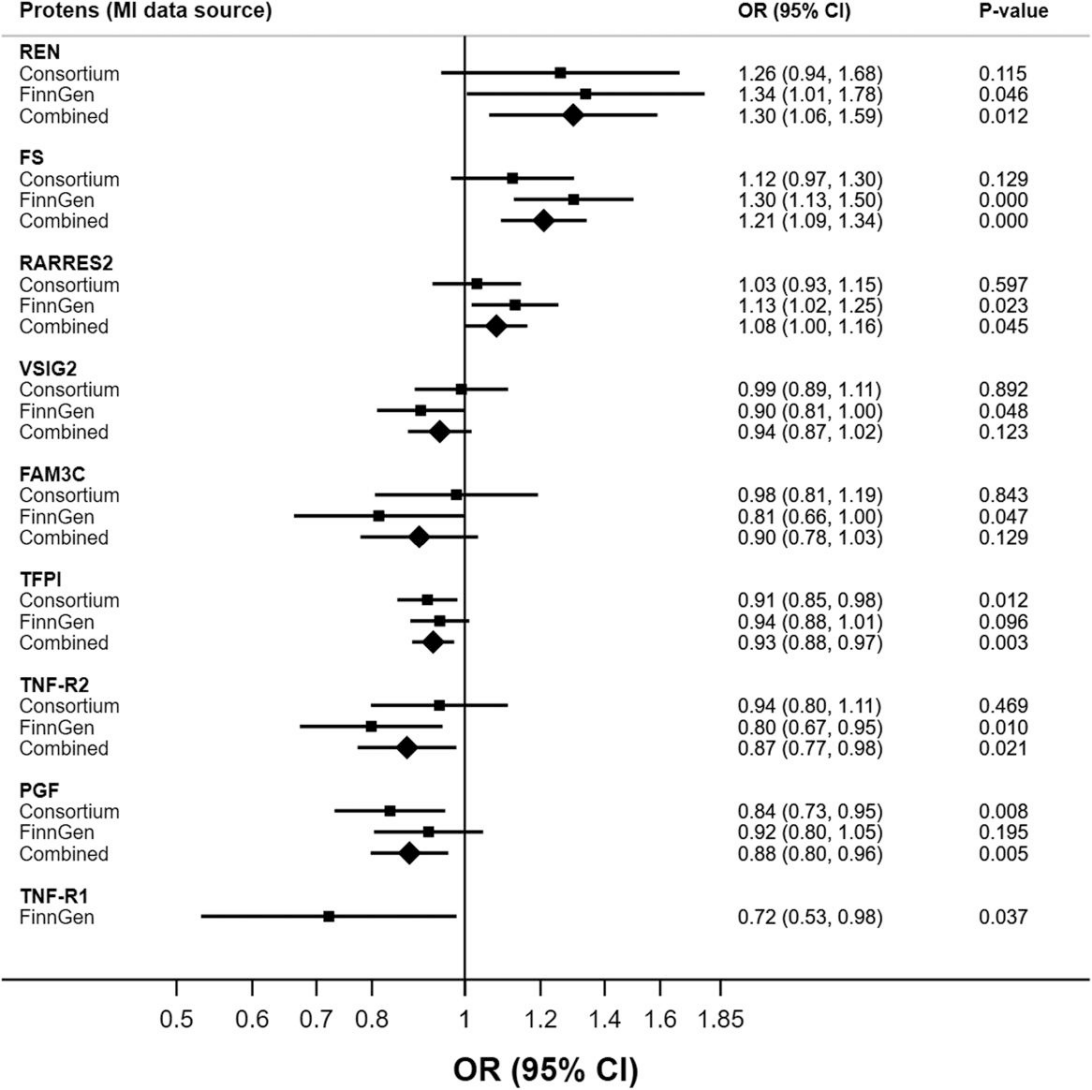
Potential causal associations of circulating proteins with the risk of myocardial infarction, Mendelian randomization analysis. OR, odds ratio; CI, confidence interval.

Meta-analysis based on the outcome data sources from CARDIoGRAMplusC4D and FinnGen revealed that higher genetically predicted levels of renin (REN), follistatin (FS), and RARRES2 were associated with an increased risk of MI, which is in line with cohort-based analyses. In contrast, genetically predicted tissue factor pathway inhibitor (TFPI) levels, TNF-R2, placenta growth factor (PGF), and TNF-R1 were inversely associated with MI. In addition, a suggestive inverse association between V-set and immunoglobulin domain-containing protein 2, VSIG2, as well as FAM3C, and the risk of MI was found in the analysis based on the FinnGen study; however, these associations were not statistically significant in the combined analysis of both cohorts (*Figure 3*, Supplementary data online, *Table S10*).

### Assessment of druggability

Six of the seven proteins potentially causally associated with MI in the meta-analysis and TNF-R1 linked to MI in FinnGen (*Figure 3*) have been targets for drug development (see Supplementary data online, *Table S12*). One drug related to REN has been approved as a renin inhibitor used to manage hypertension. Two drugs targeting TFPI have been approved as promoters of blood coagulation. PGF is one of the drug targets for vascular endothelial growth factor (VEGF) inhibitors used to treat, for example, macular edema following retinal vein occlusion, diabetic macular edema, and diabetic retinopathy. TNF-R1 and TNF-R2 are targets for drugs with indications to treat soft tissue sarcoma. No drug information was found for RARRES2.

## Discussion

Using a discovery and replication design, we have identified 45 circulating proteins associated with incident MI. Several of these proteins, such as NPDC1, CCL15, and TR-AP, have not been previously linked to the risk of MI in population-based cohort studies. Notably, several of our findings were sex-specific, with most protein-MI associations identified among women. MR analysis revealed potential causal relationships of REN, FS, RARRES2, TNF-R2, TNF-R1, PGF, and TFPI proteins with MI. However, the associations for TNF-R2, TNF-R1, PGF, and TFPI were in opposite directions in the observational and MR analyses (*Structured Graphical Abstract*).

Given the large number of proteins identified, our discussion is limited to the proteins most strongly related to incident MI in cohort-based and MR analyses, involved in common pathophysiological processes, and seldom or never reported in cohort studies. In the discovery cohort, the strongest association with an increased risk of MI was observed for GDF15, ADM, and KIM1. GDF15 is usually upregulated under episodes of stress, such as cardiac ischemia or reperfusion,^29^ and elevated blood levels of GDF15 have been related to endothelial dysfunction and the risk for incident CVDs.^10,11,30^ ADM has several functions, including vasodilation, regulation of hormone secretion and promotion of angiogenesis, and its plasma level is elevated in patients with hypertension, MI, and heart failure.^31^ KIM1 (HAVCR1) is mainly described in relation to renal dysfunction. Still, it has also been suggested as a potentially promising biomarker for early CVD diagnosis, monitoring of therapeutic effects, and prediction of clinical outcomes in patients with CVDs.^32^ Similar to our findings, higher GDF15, ADM, and KIM1 levels have been linked to an increased risk of incident MI in cohort studies.^10,13^ This suggests that these proteins are possible mediators or non-causal markers of CVD risk factors and MI. In addition, GDF15, KIM-1, and ADM were associated with overall mortality in our study.

Atherosclerosis is the leading cause of MI.^1^ We found several atherosclerosis-related proteins linked to an increased risk of MI, e.g. CTSD, SELP, CHI3L1, U-PAR, MMP-12, and MMP-3. SELP is involved in inflammatory processes and positively associates with incident CHD.^33^ In previous cohort studies, higher levels of CTSD, U-PAR, MMP-12, and CHI3L1 have been associated with the risk of incident MI.^13^ Members of the matrix metalloproteinase family, including MMP-12 and MMP-3, are involved in tissue remodeling, wound repair, and the progression of atherosclerosis.^34^ U-PAR mediates several functions of the plasminogen activation system and plays essential roles in hemostasis, fibrinolysis, and inflammation; this protein is implicated in the pathophysiology of atherosclerosis.^35^ Higher blood levels of GDF15, MMP-12, and U-PAR have been reported to be associated with worse systolic function evaluated as left ventricular ejection fraction,^36^ further suggesting a common mechanistic link to the risk of MI.

Another protein related to fibrinolysis and coagulation identified in our study is TFPI. This protein is the primary inhibitor of tissue factor (TF)-mediated coagulation and has therefore been suggested to play a role in thrombosis and atherosclerosis.^37^ We observed that higher concentrations of TFPI were associated with an increased rate of MI in our cohort-based analysis. In contrast, the opposite direction of association was found in our MR analysis. The total plasma TFPI levels include lipid-bound and free TFPI, which may exhibit different biological activity.^38^ This may partly explain the difference in our results, i.e. plasma levels reflect the concentration of free TFPI, whereas genetically predicted levels of TFPI reflect total TFPI. The opposing results may also be explained by a persisting, genetically determined procoagulant state or underlying health conditions (e.g. pro-atherogenic processes) that influence the balance of the coagulation system with feedback loops to increase TFPI. This protein is presently a target for a drug that promotes blood coagulation.

Extensive data suggest the implication of immune-inflammatory pathways in the development of atherosclerosis and associated CVDs.^10,39^ Several proteins related to the inflammatory response have been linked to the elevated risk of MI in our cohort studies, such as RARRES2, CLEC5A, TNF-R1, TNF-R2, and PGF. Thus, our findings of a robust positive association between circulating RARRES2 and MI are consistent with the previous cohort studies.^13,39^ RARRES2, also known as chemerin, is mainly expressed in adipose tissue and has been implicated in the regulation of adipogenesis, immune response regulation, glucose metabolism, and inflammation.^40^ The results of our MR analysis suggested a causal role of elevated levels of RARRES2 in the risk of MI. However, no drug information was found for this protein.

In contrast, TNF-R1, TNF-R2, and PGF had opposite directions of association in our study's cohort-based and MR analyses. TNF-R1 and TNF-R2 are members of the TNF-receptor superfamily involved in TNF-α signaling,^41^ and they are highly correlated in our study (correlation coefficient of 0.83). In line with our findings, associations between elevated levels of TNF-R1, TNF-R2, and an increased risk of MI or other CVDs have been observed in previous cohort studies.^10,13^ PGF is a member of the VEGF family implicated in immune responses, vascular homeostasis, and angiogenesis. The potential link with MI has been mainly described in the literature in patient cohorts or experimental studies.^42^ In addition, a positive association between circulating PGF levels and the risk of heart failure has been shown.^13^ An inverse association between PGF and the risk of MI in our MR analysis is in line with another recent MR.^42^ The opposite directions of association of the link between specific proteins and the risk of CVD outcomes in the cohort and MR analyses have previously been reported, for example, in relation to the risk of incident ischemic stroke or peripheral artery disease.^11,21^ The reason for this inconsistency remains unclear. Still, it may be potentially associated with the feedback mechanism.^11^ Thus, given that TNF-R1, TNF-R2, and PGF are involved in systemic inflammation and atherosclerosis,^42,43^ they may be upregulated to compensate for pro-atherogenic processes during early atherogenesis. Therefore participants with a reduced capacity to synthesize such proteins would have an increased risk of CVD outcomes, as observed in our MR analysis.^11^

In addition, in our study genetically predicted higher levels of REN and FS were linked to an increased risk of MI, which aligns with our cohort-based findings. REN is a part of the renin-angiotensin-aldosterone system involved in regulating blood pressure and electrolyte balance, and plasma REN has been previously associated with an increased risk of heart failure in conditionally healthy participants,^13^ or CHD.^44^ However, the evidence is inconsistent, and an increased risk of CHD was mainly observed in participants with hypertension.^44^ FS protein has multiple auto- and paracrine functions in different tissues,^45^ and previously linked to an increased risk of incident MI and other CVDs in cohort studies.^12,13,45^ Several drugs targeting proteins causally associated with MI in our study, except for RARRES2, have been approved by FDA or are under investigation. However, the indication is not always aimed at CVD-related outcomes.

We are unaware of previous cohort studies reporting the association between circulating NPDC1, CCL15, and TR-AP levels and the risk of MI in apparently healthy individuals. The literature does not describe the role of NPDC1 and CCL15 in developing MI or other CVDs. Our recent cohort study, though, has found a positive association between circulating CCL15 and the risk of incident peripheral artery disease.^21^ TR-AP, also called ACP5 or TRACP-5b, is involved in bone homeostasis and is a known marker of osteoporosis, which has also been associated with the severity of coronary atherosclerosis in patients with CHD.^6^

Our sex-specific analysis identified 12 proteins positively associated with incident MI in women but not men, such as C-X-C motif chemokine 16 (CXCL16), U-PAR, and fibroblast growth factor 23 (FGF-23). In addition, the association between GDF-15 and MI was more pronounced in women than men. Chemokines play a variety of roles in inflammation, immunity, injury repair, and other cellular responses, and CXCL16 has also been implicated in the progression of atherosclerosis^46^ and linked to an increased risk of MI.^13^ FGF-23 is involved in the regulation of phosphate and calcium homeostasis,^47^ and higher levels of this protein have been associated with several CVDs, including MI.^13^ Such sex-related disparities can be attributed to differences in sex hormones or sex hormone receptors, cardiovascular epigenetics,^48^ or metabolic and lifestyle risk factors in women and men. For example, a cross-sectional study of the link between sex hormones and FGF-23 revealed that a more androgenic sex hormone profile (higher endogenous free testosterone and lower sex hormone binding globulin levels) was directly associated with FGF-23 in postmenopausal women but inversely to FGF-23 levels in men free of clinical CVD at baseline.^47^ However, previous studies on sex differences in circulating proteins and risk of specific CVDs are limited,^48^ especially in healthy individuals, and the observed sex-specific difference in the relationship of specific proteins with MI risk in our cohort analyses warrant further investigation.

Compared with other studies on the link between circulating proteins and the risk of MI, novel aspects and strengths of our study include the large sample size, objectively and accurately assessed incident cases of MI, a discovery-replication approach using different non-related cohorts, simultaneous measurements of a large number of proteins, adjustment for important confounders, analyses stratified by sex, and complementary analyses using MR. Several limitations, however, apply to our analyses. The proteins in our discovery cohort were identified by targeted cardio-metabolic proteomics using three protein panels. Thus, proteins not included in these panels may also have a strong association with MI. In addition, we could not conduct specific analysis in relation to ST-elevation MI (STEMI) and non-STEMI as such information was not available in the Swedish patient register. Exploring the link between circulating proteins and specific types of acute MI could be of significant interest to future studies, given differences in pathophysiology, prognosis, and treatment options. Since the results are based on individuals of European ancestry, the generalizability of our results to other populations is unknown. Although we conducted MR analysis to explore causality, several proteins’ instrumental variables were unavailable. Therefore, future MR investigations are needed to confirm the observed associations. It could also be interesting to further investigate sex-specific associations between circulation proteins and incident MI by applying the MR approach when such genetic data becomes available.

In conclusion, our findings have revealed strong associations of 45 circulating proteins with the risk of incident MI in women and men, suggesting that atherosclerosis, thrombosis, inflammation, immune system-related pathways, injury and tissue repair, coagulation, bone homeostasis, and iron metabolism may play a role in the pathogenesis of MI. Several identified proteins had potential causal associations with MI based on MR analyses. Moreover, we have explored and identified several proteins linked to MI in a sex-specific manner, highlighting the importance of including both women and men in clinical and pre-clinical studies and further investigating sex differences in protein–disease relationships.

## Supplementary Material

ehae658_Supplementary_Data

## Declarations

### Data Availability

The data that support findings of this study are available upon application to the Swedish Infrastructure for Medical Population-based Life-course Environmental Research (SIMPLER; https://www.simpler4health.se). Data used for the MR analyses are based on publicly available summary statistics data provided by genetic consortia.
