# Supplementary material for: Plasma proteome and incident myocardial infarction: sex-specific differences
Source: Eur Heart J. 2024 Oct 14;45(43):4647–57. doi: 10.1093/eurheartj/ehae658 (PMC11560279; doi:10.1093/eurheartj/ehae658)

Supplementary Material

Plasma proteome and incident myocardial infarction: sex-specific differences

Olga E. Titova, Shuai Yuan, Liisa Byberg, John A. Baron, Lars Lind, Karl Michaëlsson and Susanna C. Larsson

Content	*Page*
**Supplementary information.** Methods	2-4
**Supplementary Table 1.** Proteins included in the CVD II, CVD III and Metabolism assay Olink panels in SIMPLER cohorts	5-10
**Supplementary Table 2.** Proteins in the CVD II, CVD III and Metabolism panels excluded from the analysis based on SIMPLER (more than 50% of samples were below LOD)	11
**Supplementary Table 3.** Details of the single nucleotide polymorphisms used as instrumental variables in the Mendelian randomization analysis	12-14
**Supplementary Table 4.** Baseline characteristics of SIMPLER participants by the incident myocardial infarction (MI) status during follow-up	15
**Supplementary Table 5.** Baseline characteristics of the study participants in the replication cohort **UK Biobank**	16
**Supplementary Table 6.** Associations between replicated proteins and incident myocardial infarction in the entire **SIMPLER cohort** in fully adjusted analyses, n=11,751	17-18
**Supplementary Table 7.** Associations between replicated proteins and incident myocardial infarction in the **UK Biobank cohort** in fully adjusted analyses, n=51,613	19-20
**Supplementary Table 8.** Associations between circulating proteins and incident myocardial infarction in **women** in the SIMPLER cohort and the UK Biobank cohort	21-22
**Supplementary Table 9.** Associations between circulating proteins and incident myocardial infarction in **men** in the SIMPLER cohort and the UK Biobank cohort	23-24
**Supplementary Table 10.** Results of Mendelian randomization analysis for identified protein-related SNPs and the risk of myocardial infarction	25-28
**Supplementary Table 11.** Associations between 45 proteins identified in the primary analysis and overall mortality in SIMPLER in fully adjusted analyses (n = 12,314)	29-30
**Supplementary Table 12.** Druggability of proteins associated with MI in Mendelian randomization analysis	31-32
**Supplementary Figure 1.** Flow chart and study design based on SIMPLER and UK Biobank cohorts and Mendelian randomization analysis.	33
**Supplementary Figure 2.** Correlation between 45 replicated circulating proteins associated with incident myocardial infarction in the analysis based on SIMPLER.	34

Supplementary Material

Supplementary information. Methods

1. **SIMPLER (discovery sample)**

1.1. Cohort description

Participants included in SIMPLER subcohorts (referred to here as the *Uppsala cohort* and *Västmanland cohort*) were randomly selected from the two larger population-based longitudinal SIMPLER cohorts, the Swedish Mammography Cohort (SMC) and the Cohort of Swedish Men (COSM), which were designed to explore the link between lifestyle, dietary exposures, and disease outcomes. Briefly, the SMC was established between 1987 and 1990, when all women living in Västmanland and Uppsala counties and born between 1914 and 1948 received a questionnaire on dietary habits and other characteristics (90,303 women invited, 74% response rate). The COSM was established in 1997 and included men living in Västmanland and Örebro counties, born between 1918 and 1952 (100,303 men invited, 49% response rate). In 1997, the 56,030 participants in SMC who were still alive and resided in the study area received an expanded diet and lifestyle questionnaire similar to one sent to COSM participants (70% response rate).[1] In 2008, a new *Health questionnaire* was sent to the participants in SMC and COSM who were alive (response rate: 63% in SMC and 78% in COSM). In 2009, SMC and COSM participants who completed the health questionnaire in 2008 received an updated *Diet and lifestyle questionnaire* (response rate: 84% in SMC and 90% in COSM).[2] The questionnaires were identical besides specific questions relevant for women or men (e.g., related to the age of menopause or problems with urination). According to previous research based on these cohorts, SMC and COSM are representative of the Swedish population in terms of age distribution, educational level, and prevalence of overweight and obesity. [3]

Between November 2003 and October 2009, the 5022 women who were randomly chosen for the subcohort named *Uppsala cohort* underwent a clinical examination that included dual-energy X-ray absorptiometry (DXA) measurements, blood and urine samples, fat biopsies, and anthropometric assessment. In 2010–2019, women and men living in Västmanland County who participated earlier in SMC or COSM were invited to take part in a clinical investigation similar to the one described for the Uppsala cohort (referred to as the *Västmanland cohort*). The questionnaire filled out close to the clinical examination as well as other measurements were similar. The harmonization of the questionnaire-based data and clinical or biochemical parameters was done prior to the imputation of missing values.

Proteomic analysis

Supplementary Material

Venous blood samples were collected after a 12-hour overnight fast, and the samples were immediately centrifuged and stored at −80°C until analysis. The analyses were performed at SciLifeLab, Uppsala University, Sweden. Inter-plate variability was adjusted for by intensity normalization with the plate median as the normalization factor. For data analysis Olink NPX Manager Software was used. In addition, *bridge sample normalization* using 100 bridging samples was applied by Olink Bioscience to reduce any technical variation in the results of two cohorts (www.olink.com).

N-terminal prohormone brain natriuretic peptide (NT-proBNP) was measured in both the CVD III and Metabolism panels. The latter was used for the analyses since more values passed the internal quality controls there.

1.2. Assessment of covariates

Physical activity was reported as walking or bicycling and leisure time exercise. The physical activity questionnaire has been validated using 7-day activity records and accelerometer data.[4] Potential confounders were selected using directed acyclic graphs[5] based on our *a priori* knowledge of the relationships among potential confounders, intermediate variables, exposure, and outcome variables, as well as on existing information regarding factors associated with risk of MI and circulating proteins. Plasma glucose, LDL- and HDL-cholesterol levels were measured with standard analytical methods.

2. UK Biobank (replication sample)

2.1. Proteomic analysis

Plasma levels of 2,923 proteins were measured by the Olink proteomics assay Explore (OLINK, Uppsala, Sweden) using the proximity extension assay technique. An extensive quality control was conducted, and according to the information from the manufacturer, the observed mean intra-assay and inter-assay coefficients of variation were in the 8-17% range. The proteomic analyses in UK Biobank have been previously described.[6] Data on proteins METRNL and MMP-2 was not available in the UK Biobank sample used for the replication analyses. Abbreviations and names for some proteins differed between SIMPLER and UK Biobank samples. For simplicity, the same abbreviations and names given for SIMPLER cohorts were used in all results presented based on SIMPLER and UK Biobank.

Supplementary Material

2.2. Assessment of covariates

Serum glucose, LDL- and HDL-cholesterol concentrations were assessed by a Beckman coulter AU5800, by standard methods. Blood pressure was measured twice in the sitting position with an automated Omron device. Ethnicity was categorized into four groups: White, Black, Asian, and other. Townsend social deprivation index was used as a proxy of socioeconomic status. Smoking was categorized as never, previous or current.

References:

1. Titova OE, Baron JA, Michaëlsson K, Larsson SC. Anger frequency and risk of cardiovascular morbidity and mortality. Eur Heart J Open. 2022 Aug 6;2(4):oeac050.

2. Harris H, Håkansson N, Olofsson C, Stackelberg O, Julin B, Åkesson A, Wolk A. The Swedish mammography cohort and the cohort of Swedish men: study design and characteristics of 2 population-based longitudinal cohorts. OA Epidemiol 2013;1:16.

3. Stackelberg O, Bjorck M, Larsson SC, Orsini N, Wolk A. Sex differences in the association between smoking and abdominal aortic aneurysm. Br J Surg. Sep 2014;101(10):1230-7. doi:10.1002/bjs.9526

4. Orsini N, Bellocco R, Bottai M, Hagstromer M, Sjostrom M, Pagano M, et al. Validity of self-reported total physical activity questionnaire among older women. Eur J Epidemiol. 2008;23(10):661-7.

5. Textor J and Hardt J. DAGitty: A graphical tool for analyzing causal diagrams. *Epidemiology*. 2011; 22:745.

6. Sun BB, Chiou J, Traylor M, Benner C, Hsu Y-H, Richardson TG, et al. Plasma proteomic associations with genetics and health in the UK Biobank. *Nature* 2023; 622(7982):329-338.

Supplementary Material

Supplementary Table 1. Proteins included in the CVD II, CVD III and Metabolism assay Olink panels in SIMPLER cohorts

CVD II panel		CVD III panel		Metabolism panel	
**Abbreviation**	**Name**	**Abbreviation**	**Name**	**Abbreviation**	**Name**
ACE2	Angiotensin-converting enzyme 2	ALCAM	CD166 antigen	AHCY	Adenosylhomocysteinase
ADAM-TS13	A disintegrin and metalloproteinase withthrombospondin motifs 13	AP-N	Aminopeptidase N	ADGRE2	Adhesion G protein-coupled receptor E2
ADM	Adrenomedullin	AXL	Tyrosine-protein kinase receptor UFO	ADGRG2	Adhesion G-protein coupled receptor G2
AGRP	Agouti-related protein	AZU1	Azurocidin	APLP1	Amyloid-like protein 1
AMBP	Protein AMBP	BLM hydrolase	Bleomycin hydrolase	ANGPT2	Angiopoietin-2
ANGPT1	Angiopoietin-1	CASP-3	Caspase-3	ANGPTL1	Angiopoietin-related protein 1
BMP-6	Bone morphogenetic protein 6	CCL15	C-C motif chemokine 15	ANGPTL7	Angiopoietin-related protein 7
BNP	Natriuretic peptides B	CCL16	C-C motif chemokine 16	ANXA11	Annexin A11
CA5A	Carbonic anhydrase 5A, mitochondrial	CCL22	C-C motif chemikine 22	ANXA4	Annexin A4
CCL17	C-C motif chemokine 17	CCL24	C-C motif chemokine 24	GHRL	Appetite-regulating hormone
CCL3	C-C motif chemokine 3	CD163	Scavenger receptor cysteine-rich type 1 protein M130	ARG1	Arginase-1
CD4	T-cell surface glycoprotein CD4	CD93	Complement component C1q receptor	DDC	Aromatic-L-amino-acid decarboxylase
CD40-L	CD40 ligand	CDH5	Cadherin-5	CD79B	B-cell antigen receptor complex-associated protein beta chain
CD84	SLAM family member 5	CHI3L1	Chitinase-3-like protein 1	CDH2	Cadherin-2

Supplementary Material

CEACAM8	Carcinoembryonic antigenrelated cell adhesion molecule 8	CHIT1	Chitotriosidase-1	CDHR5	Cadherin-related family member 5
CTRC	Chymotrypsin C	CNTN1	Contactin-1	CLSTN2	Calsyntenin-2
CTSL1	Cathepsin L1	COL1A1	Collagen alpha-1(I) chain	CA13	Carbonic anhydrase 13
CXCL1	C-X-C motif chemokine 1	CPA1	Carboxypeptidase A1	COMT	Catechol O-methyltransferase
DCN	Decorin	CPB1	Carboxypeptidase B	CTSO	Cathepsin O
DECR1	2,4-dienoyl-CoA reductase, mitochondrial	CSTB	Cystatin-B	CD2AP	CD2-associated protein
Dkk-1	Dickkopf-related protein 1	CTSD	Cathepsin D	CHRDL2	Chordin-like protein 2
FABP2	Fatty acid-binding protein, intestinal	CTSZ	Cathepsin Z	CLUL1	Clusterin-like protein 1
FGF21	Fibroblast growth factor 21	CXCL16	C-X-C motif chemokine 16	CCDC80	Coiled-coil domain-containing protein 80
FGF-23	Fibroblast growth factor 23	DLK-1	Protein delta homolog 1	CRKL	Crk-like protein
FS	Follistatin	EGFR	Epidermal growth factor receptor	CLEC5A	C-type lectin domain family 5 member A
Gal-9	Galectin-9	Ep-CAM	Epithelial cell adhesion molecule	CLMP	CXADR-like membrane protein
GDF2	Growth differentiation factor 2	EPHB4	Ephrin type-B receptor 4	DIABLO	Diablo homolog, mitochondrial
GH	Growth hormone	FABP4	Fatty acid-binding protein, adipocyte	QDPR	Dihydropteridine reductase
GIF	Gastric intrinsic factor	FAS	Tumor necrosis factor receptor superfamily member 6	DPP7	Dipeptidyl peptidase 2
GLO1	Lactoylglutathione lyase	Gal-3	Galectin-3	DAB2	Disabled homolog 2
GT	Gastrotropin	Gal-4	Galectin-4	APEX1	DNA-(apurinic or apyrimidinic site) lyase

Supplementary Material

HAOX1	Hydroxyacid oxidase 1	GDF15	Growth differentiation factor 15	ENTPD5	Ectonucleoside triphosphate diphosphohydrolase 5
HB-EGF	Proheparin-binding EGF-like growth factor	GRN	Granulins	ENPP7	Ectonucleotide pyrophosphatase/phosphodiesterase family member 7
HO-1	Heme oxygenase 1	ICAM-2	Intercellular adhesion molecule 2	RNASE3	Eosinophil cationic protein
hOSCAR	Osteoclast-associated immunoglobulin-like receptor	IGFBP-1	Insulin-like growth factor-binding protein 1	FCRL1	Fc receptor-like protein 1
HSP 27	Heat shock 27 kDa protein	IGFBP-2	Insulin-like Growth Factor-Binding Protein 2	FBP1	Fructose-1,6-bisphosphatase 1
IDUA	Alpha-L-iduronidase	IGFBP-7	Insulin-like growth factor-binding protein 7	GAL	Galanin peptides
IgG Fc receptor II-b	Low affinity immunoglobulin gamma Fc region receptor II-b	IL-17RA	Interleukin-17 receptor A	ENO2	Gamma-enolase
IL16	Pro-interleukin-16	IL-18BP	Interleukin-18-binding protein	GLRX	Glutaredoxin-1
IL-17D	Interleukin-17D	IL-1RT1	Interleukin-1 receptor type 1	GRAP2	GRB2-related adapter protein 2
IL-18	Interleukin-18	IL-1RT2	Interleukin-1 receptor type 2	HDGF	Hepatoma-derived growth factor
IL-1ra	Interleukin-1 receptor antagonist protein	IL2RA	Interleukin-2 receptor subunit alpha	ROR1	Inactive tyrosine-protein kinase transmembrane receptor ROR1
IL1RL2	Interleukin-1 receptor-like 2	IL-6RA	Interleukin-6 receptor subunit alpha	IGFBPL1	Insulin-like growth factor-binding protein-like 1
IL-27	Interleukin-27	ITGB2	Integrin beta-2	ITGB7	Integrin beta-7
IL-4RA	Interleukin-4 receptor subunit alpha	JAM-A	Junctional adhesion molecule A	KLK10	Kallikrein-10
IL-6	Interleukin-6	KLK6	Kallikrein-6	KYAT1	Kynurenine--oxoglutarate transaminase 1

Supplementary Material

ITGB1BP2	Melusin	LDL receptor	Low-density lipoprotein receptor	BAG6	Large proline-rich protein BAG6
KIM-1	Kidney injury molecule 1	LTBR	Lymphotoxin-beta receptor	LRIG1	Leucine-rich repeats and immunoglobulin-like domains protein 1
LEP	Leptin	MB	Myoglobin	LILRA5	Leukocyte immunoglobulin-like receptor subfamily A member 5
LOX-1	Lectin-like oxidized LDL receptor 1	MCP-1	Monocyte chemotactic protein 1	LRP11	Low-density lipoprotein receptor-related protein 11
LPL	Lipoprotein lipase	MEPE	Matrix extracellular phosphoglycoprotein	ACP6	Lysophosphatidic acid phosphatase type 6
MARCO	Macrophage receptor MARCO	MMP-2	Matrix metalloproteinase-2	MEP1B	Meprin A subunit beta
MERTK	Tyrosine-protein kinase Mer	MMP-3	Matrix metalloproteinase-3	METRNL	Meteorin-like protein
MMP-12	Matrix metalloproteinase-12	MMP-9	Matrix metalloproteinase-9	MCFD2	Multiple coagulation factor deficiency protein 2
MMP-7	Matrix metalloproteinase-7	MPO	Myeloperoxidase	NADK	NAD kinase
NEMO	NF-kappa-B essential modulator	Notch 3	Neurogenic locus notch homolog protein 3	NECTIN2	Nectin-2
PAPPA	Pappalysin-1	NT-pro BNP	N-terminal prohormone brain natriuretic peptide	NPDC1	Neural proliferation differentiation and control protein 1
PAR-1	Proteinase-activated receptor 1	OPG	Osteoprotegerin	NPTXR	Neuronal pentraxin receptor
PARP-1	Poly [ADP-ribose] polymerase 1	OPN	Osteopontin	NOMO1	Nodal modulator 1
PDGF subunit B	Platelet-derived growth factor subunit B	PAI	Plasminogen activator inhibitor 1	NT-proBNP	N-terminal prohormone of brain natriuretic peptide
PD-L2	Programmed cell death 1 ligand 2	PCSK9	Proprotein convertase subtilisin/kexin type 9	PILRB	Paired immunoglobulin-like type 2 receptor beta
PIgR	Polymeric immunoglobulin receptor	PDGF subunit A	Platelet-derived growth factor subunit A	FKBP4	Peptidyl-prolyl cis-trans isomerase FKBP4

Supplementary Material

PGF	Placenta growth factor	PECAM-1	Platelet endothelial cell adhesion molecule	PAG1	Phosphoprotein associated with glycosphingolipid- enriched microdomains 1
PRELP	Prolargin	PGLYRP1	Peptidoglycan recognition protein 1	CTSH	Pro-cathepsin H
Protein BOC	Brother of CDO	PI3	Elafin	FAM3C	Protein FAM3C
PRSS27	Serine protease 27	PLC	Perlecan	PPP1R2	Protein phosphatase inhibitor 2
PRSS8	Prostasin	PON3	Paraoxonase (PON 3)	S100P	Protein S100-P
PSGL-1	P-selectin glycoprotein ligand 1	PRTN3	Myeloblastin	REG4	Regenerating islet-derived protein 4
PTX3	Pentraxin-related protein PTX3	PSPD	Pulmonary surfactant-associated protein D	RTN4R	Reticulon-4 receptor
RAGE	Receptor for advanced glycosylation end products	RARRES2	Retinoic acid receptor responder protein 2	ALDH1A1	Retinal dehydrogenase 1
REN	Renin	RETN	Resistin	NQO2	Ribosyldihydronicotinamide dehydrogenase [quinone]
SCF	Stem cell factor	SCGB3A2	Secretoglobin family 3A member 2	SSC4D	Scavenger receptor cysteine-rich domain-containing group B protein
SERPINA12	Serpin A12	SELE	E-selectin	SOST	Sclerostin
SLAMF7	SLAM family member 7	SELP	P-selectin	SEMA3F	Semaphorin-3F
SOD2	Superoxide dismutase [Mn], mitochondrial	SHPS-1	Tyrosine-protein phosphatase non- receptor type substrate 1	SERPINB6	Serpin B6
SORT1	Sortilin	SPON1	Spondin-1	SERPINB8	Serpin B8
SPON2	Spondin-2	ST2	ST2 protein	SIGLEC7	Sialic acid-binding Ig-like lectin 7

Supplementary Material

SRC	Proto-oncogene tyrosine-protein kinase Src	TFF3	Trefoil factor 3	CD164	Sialomucin core protein 24
STK4	Serine/threonine-protein kinase 4	TFPI	Tissue factor pathway inhibitor	CANT1	Soluble calcium-activated nucleotidase 1
TF	Tissue factor	TIMP4	Metalloproteinase inhibitor 4	SUMF2	Sulfatase-modifying factor 2
TGM2	Protein-glutamine gamma-glutamyltransferase 2	TLT-2	Trem-like transcript 2 protein	SNAP23	Synaptosomal-associated protein 23
THBS2	Thrombospondin-2	TNF-R1	Tumor necrosis factor receptor 1	SDC4	Syndecan-4
THPO	Thrombopoietin	TNF-R2	Tumor necrosis factor receptor 2	CD1C	T-cell surface glycoprotein CD1c
TIE2	Angiopoietin-1 receptor	TNFRSF10C	Tumor necrosis factor receptor superfamily member 10C	THOP1	Thimet oligopeptidase
TM	Thrombomodulin	TNFRSF14	Tumor necrosis factor receptor superfamily member 14	TXNDC5	Thioredoxin domain-containing protein 5
TNFRSF10A	Tumor necrosis factor receptor superfamily member 10A	TNFSF13B	Tumor necrosis factor ligand superfamily member 13B	TYMP	Thymidine phosphorylase
TNFRSF11A	Tumor necrosis factor receptor superfamily member 11A	t-PA	Tissue-type plasminogen activator	TSHB	Thyrotropin subunit beta
TNFRSF13B	Tumor necrosis factor receptor superfamily member 13B	TR	Transferrin receptor protein 1	TFF2	Trefoil factor 2
TRAIL-R2	TNF-related apoptosis-inducing ligand receptor 2	TR-AP	Tartrate-resistant acid phosphatase type 5	TINAGL1	Tubulointerstitial nephritis antigen-like
VEGF-D	Vascular endothelial growth factor D	uPA	Urokinase-type plasminogen activator	TYRO3	Tyrosine-protein kinase receptor TYRO3
VSIG2	V-set and immunoglobulin domain-containing protein 2	U-PAR	Urokinase plasminogen activator surface receptor	USP8	Ubiquitin carboxyl-terminal hydrolase 8
XCL1	Lymphotactin	vWF	von Willebrand factor	VCAN	Versican core protein

Supplementary Material

Supplementary Table 2. Proteins in the CVD II, CVD III and Metabolism panels excluded from the analysis based on SIMPLER (more than 50% of samples were below LOD)

Uppsala and Västmanland cohorts			
**CVD II panel**	**CVD III panel**	**Metabolism panel**	
PAPPA	CCL22	AHCY	CTSH
PARP-1		S100P	NQO2
		GLRX	DPP7
		DIABLO	ARG1
		ANXA4	USP8
		ANXA11	FKBP4
		ITGB7	DAB2

Abbreviations of proteins can be found in **Supplementary Table 1**.

Supplementary Material

Supplementary Table 3. Details of the single nucleotide polymorphisms used as instrumental variables in the Mendelian randomization analysis

Protein abbreviation	Alternative abbreviation	SNP	Effect allele	Other allele	Beta	SE	P value
**Outcome data source – *Nikpay et al. 2015, CARDIoGRAMplusC4D Consortium, sample size =171,875***							
LILRA5		rs11084329	C	T	-0.300	0.008	1.00E-200
VSIG2		rs11219769	T	G	0.213	0.008	1.20E-142
MMP-12		rs117563322	T	C	-0.608	0.016	1.00E-200
TR-AP	ACP5	rs12983092	T	C	0.335	0.011	8.22E-197
Gal-4	LGALS4	rs140695578	T	A	0.280	0.019	3.68E-47
CHI3L1		rs1538372	G	A	0.295	0.008	1.00E-200
TFPI		rs17464221	T	C	0.308	0.008	1.00E-200
U-PAR	PLAUR	rs2302524	C	T	-0.177	0.010	1.55E-74
TR	TFRC	rs2343851	A	T	-0.407	0.012	1.00E-200
TNFRSF14		rs2495366	A	G	0.115	0.007	3.86E-54
FAM3C		rs2707502	A	T	-0.114	0.008	4.78E-43
TRAIL-R2	TNFRSF10B	rs2889	G	A	0.310	0.008	1.00E-200
MMP-3	MMP3	rs3020919	T	C	0.257	0.007	1.00E-200
FS	FST	rs31226	C	T	-0.142	0.008	1.89E-77
RARRES2		rs3735167	T	C	0.216	0.008	3.27E-144
IGFBP-1		rs3828998	C	T	-0.047	0.008	6.86E-10
ADM		rs4267063	T	G	0.153	0.007	3.71E-103
OPG	TNFRSF11B	rs4629902	C	A	0.080	0.007	6.17E-28
KIM1	HAVCR1	rs4704836	A	G	0.440	0.007	1.00E-200
CTSZ		rs4764822	T	C	-0.319	0.008	1.00E-200
REN		rs4951315	A	C	-0.076	0.008	2.08E-22
OPN	SPP1	rs56254643	C	T	-0.114	0.009	7.26E-36
CTSD		rs56385468	G	T	-0.511	0.013	1.00E-200
TNF-R2	TNFRSF1B	rs5746026	A	G	-0.392	0.019	1.17E-96

SELP		rs6136	G	T	-0.403	0.012	1.00E-200
FGF-23		rs6489536	C	G	0.108	0.008	1.24E-43
PGF		rs6574205	C	G	-0.153	0.007	2.84E-114
TNFRSF10A		rs67902577	C	A	0.353	0.008	1.00E-200
CLEC5A		rs6962760	C	T	0.215	0.007	1.65E-193
GDF15		rs71355881	A	C	0.368	0.010	1.00E-200
CD1C		rs76291980	T	C	-0.181	0.020	8.09E-20
CXCL16		rs78086290	T	A	0.189	0.007	1.45E-148
CCL15		rs79061680	G	T	0.090	0.016	2.78E-08
PRSS8		rs889555	T	C	0.115	0.008	5.71E-49
TIMP4		rs900138	G	A	0.322	0.008	1.00E-200
**Outcome data source – *FinnGen R10, sample size =369,139***							
LILRA5		rs11084329	C	T	-0.300	0.008	1.00E-200
VSIG2		rs11219769	T	G	0.213	0.008	1.20E-142
MMP-12		rs117563322	T	C	-0.608	0.016	1.00E-200
TR-AP	ACP5	rs12983092	T	C	0.335	0.011	8.22E-197
Gal-4	LGALS4	rs140695578	T	A	0.280	0.019	3.68E-47
CHI3L1		rs1538372	G	A	0.295	0.008	1.00E-200
TFPI		rs17464221	T	C	0.308	0.008	1.00E-200
U-PAR	PLAUR	rs2302524	C	T	-0.177	0.010	1.55E-74
TR	TFRC	rs2343851	A	T	-0.407	0.012	1.00E-200
TNFRSF14		rs2495366	A	G	0.115	0.007	3.86E-54
FAM3C		rs2707502	A	T	-0.114	0.008	4.78E-43
TRAIL-R2	TNFRSF10B	rs2889	G	A	0.310	0.008	1.00E-200
MMP-3	MMP3	rs3020919	T	C	0.257	0.007	1.00E-200
FS	FST	rs31226	C	T	-0.142	0.008	1.89E-77
RARRES2		rs3735167	T	C	0.216	0.008	3.27E-144
IGFBP-1		rs3828998	C	T	-0.047	0.008	6.86E-10

TNF-R1	TNFRSF1A	rs4149584	T	C	-0.303	0.028	5.78E-27
ADM		rs4267063	T	G	0.153	0.007	3.71E-103
OPG	TNFRSF11B	rs4629902	C	A	0.080	0.007	6.17E-28
KIM1	HAVCR1	rs4704836	A	G	0.440	0.007	1.00E-200
CTSZ		rs4764822	T	C	-0.319	0.008	1.00E-200
REN		rs4951315	A	C	-0.076	0.008	2.08E-22
OPN	SPP1	rs56254643	C	T	-0.114	0.009	7.26E-36
CTSD		rs56385468	G	T	-0.511	0.013	1.00E-200
Gal-9	LGALS9	rs571052714	G	T	-0.250	0.024	9.34E-26
TNF-R2	TNFRSF1B	rs5746026	A	G	-0.392	0.019	1.17E-96
SELP		rs6136	G	T	-0.403	0.012	1.00E-200
FGF-23		rs6489536	C	G	0.108	0.008	1.24E-43
PGF		rs6574205	C	G	-0.153	0.007	2.84E-114
TNFRSF10A		rs67902577	C	A	0.353	0.008	1.00E-200
CLEC5A		rs6962760	C	T	0.215	0.007	1.65E-193
CD1C		rs76291980	T	C	-0.181	0.020	8.09E-20
CXCL16		rs78086290	T	A	0.189	0.007	1.45E-148
CCL15		rs79061680	G	T	0.090	0.016	2.78E-08
REG4		rs79795228	A	C	0.633	0.028	2.80E-115
PRSS8		rs889555	T	C	0.115	0.008	5.71E-49

Abbreviations: EA, effect allele; EAF, effect allele frequency; SE, standard error; SNP, single nucleotide polymorphism. Outcome data source – Nikpay et al. 2015, CARDIoGRAMplusC4D Consortium or FinnGen R10

Supplementary Material

Supplementary Table 4. Baseline characteristics of SIMPLER participants by the incident myocardial infarction (MI) status during follow-up

Characteristics*	All	Without MI	Incident MI
**Number of participants**	11,751	11,349	402
**Age at baseline**, years, *means (SD)*	70.9 (6.8)	70.74 (6.7)	74.10 (7.0)
**Women**	7,423 (63.2)	7,208 (63.5)	215 (53.5)
**Education** > 12 years, *n (%)*	3,340 (29.1)	3,243 (29.3)	97 (24.4)
**Current cigarette smoking**, *n (%)**	3,457 (31.4)	3,289 (30.9)	168 (44.6)
**Alcohol intake**, g/day, *median (Q1-Q3)*	5.0 (1.4-10.8)	5.0 (1.5-10.8)	3.7 (0.6-9.7)
**Walking/bicycling** >40 min/day, *n (%)*	4,206 (38.4)	4,085 (38.6)	121 (33.5)
**Exercise** ≥ 2 hours/week, *n (%)*	4,195 (38.4)	4,079 (38.6)	116 (32.6)
**mDASH Diet Score**, *means (SD)*	18.2 (3.7)	18.2 (3.7)	17.4 (3.7)
**Systolic blood pressure**, mmHg, *means (SD)*	140.4 (17.8)	140.2 (17.8)	146.6 (18.2)
**Fasting glucose**, mmol/L, *means (SD)*	5.8 (1.5)	5.7 (1.5)	6.0 (1.5)
**LDL-C**, mmol/L, *means (SD)*	3.3 (1.0)	3.3 (1.0)	3.4 (1.0)
**HDL-C**, mmol/L, *means (SD)*	1.5 (0.4)	1.6 (0.4)	1.4 (0.3)
**Body mass index**, kg/m^2, *means (SD)*	26.3 (4.1)	26.3 (4.1)	26.8 (4.0)

Abbreviations: mDASH, modified Dietary Approaches to Stop Hypertension; LDL-C, low-density lipoprotein cholesterol; HDL-C, high density lipoprotein cholesterol; Q1 - lower quartile, Q3 - upper quartile. * Current smoking included smoking occasionally or regularly

Supplementary Material

Supplementary Table 5. Baseline characteristics of the study participants in the replication cohort **UK Biobank**

**Characteristics**	**All**	**Women**	**Men**
**Number of participants**	51,613	28,313	23,300
**Age at baseline**, years, *means (SD)*	57.2 (8.2)	57.1 (8.1)	57.3 (8.3)
**Ethnic background**, *n (%)*			
White	48,292 (93.7)	26,530 (93.8)	21,762 (93.5)
Black	1,341 (2.6)	748 (2.7)	593 (2.6)
Asian	1,193 (2.3)	590 (2.1)	603 (2.6)
Other	727 (1.4)	411 (1.5)	316 (1.4)
**Townsend social deprivation index,** mean (SD)	-1.20 (3.2)	-1.2 (3.1)	-1.2 (3.2)
**Cigarette smoking**, *n (%)*			
Never	28,404 (55.0)	16,902 (59.7)	11,502 (49.4)
Former	17,753 (34.4)	8,869 (31.3)	8,884 (38.1)
Current	5,396 (10.5)	2,508 (8.9)	2,888 (12.4)
**Prevalent diabetes mellitus**, *n (%)**	2,717 (5.3)	1,107 (3.9)	1,610 (6.9)
**Systolic blood pressure**, mmHg, *means (SD)*	139.7 (19.7)	137.1 (20.3)	142.8 (18.5)
**Body mass index**, kg/m^2, *means (SD)*	27.4 (4.8)	27.1 (5.2)	27.8 (4.2)
**LDL-C**, mmol/L, *means (SD)*	3.6 (0.9)	3.6 (0.9)	3.5 (0.9)
**HDL-C**, mmol/L, *means (SD)*	1.5 (0.4)	1.6 (0.4)	1.3 (0.3)
**Incident MI during follow-up**, *n*	1355	477	878

Low-density lipoprotein cholesterol; HDL-C, high density lipoprotein cholesterol
* Self-reported diabetes mellitus

Supplementary Material

Supplementary Table 6. Associations between replicated proteins and incident myocardial infarction in the entire **SIMPLER cohort** in fully adjusted analyses, n=11,751

	Protein abbreviation	HR	Min 95%	Max 95%	P-value
1	GDF15	1.418	1.270	1.584	6.20E-10
2	ADM	1.332	1.164	1.524	3.10E-05
3	KIM1	1.284	1.160	1.422	1.30E-06
4	TNFRSF11A	1.256	1.122	1.407	7.70E-05
5	PGF	1.247	1.101	1.412	5.20E-04
6	Gal-9	1.238	1.086	1.410	1.30E-03
7	METRNL	1.237	1.093	1.399	7.30E-04
8	AMBP	1.230	1.082	1.399	1.60E-03
9	TNFRSF10A	1.229	1.097	1.376	3.60E-04
10	OPG	1.226	1.107	1.359	9.60E-05
11	PRSS8	1.225	1.066	1.408	4.30E-03
12	CHI3L1	1.220	1.102	1.350	1.20E-04
13	IGFBP-1	1.220	1.086	1.370	8.20E-04
14	CTSL1	1.215	1.080	1.367	1.20E-03
15	VSIG2	1.212	1.091	1.348	3.60E-04
16	U-PAR	1.212	1.103	1.332	6.80E-05
17	RARRES2	1.206	1.080	1.347	9.00E-04
18	TNF-R1	1.206	1.085	1.341	5.40E-04
19	IL6	1.203	1.097	1.318	8.20E-05
20	TFPI	1.203	1.076	1.344	1.10E-03
21	FS	1.197	1.077	1.330	8.80E-04
22	Gal-4	1.192	1.073	1.323	1.00E-03
23	FGF-23	1.191	1.090	1.302	1.20E-04
24	MMP-3	1.190	1.061	1.336	3.10E-03
25	REN	1.190	1.061	1.334	2.90E-03
26	CTSZ	1.188	1.063	1.328	2.40E-03

27	CTSD	1.183	1.074	1.303	6.30E-04
28	LILRA5	1.181	1.054	1.323	4.00E-03
29	CLEC5A	1.179	1.066	1.303	1.30E-03
30	TNFRSF14	1.177	1.063	1.303	1.70E-03
31	FAM3C	1.175	1.053	1.312	4.10E-03
32	NPDC1	1.174	1.058	1.302	2.40E-03
33	OPN	1.173	1.060	1.298	1.90E-03
34	CCL15	1.166	1.059	1.283	1.70E-03
35	CXCL16	1.165	1.054	1.287	2.70E-03
36	MMP-12	1.164	1.045	1.296	5.90E-03
37	TR	1.162	1.054	1.281	2.60E-03
38	REG4	1.159	1.051	1.279	3.20E-03
39	SELP	1.156	1.050	1.272	3.20E-03
40	TFF3	1.155	1.053	1.266	2.30E-03
41	TNF-R2	1.150	1.043	1.267	5.00E-03
42	TRAIL-R2	1.147	1.059	1.242	7.30E-04
43	TIMP4	1.140	1.034	1.257	8.80E-03
44	TR-AP	1.119	1.028	1.218	9.20E-03
45	CD1C	0.813	0.736	0.897	4.30E-05

Hazard ratios (HR) with 95% confidence intervals (CI) are expressed per standard deviation unit change in protein measurements. The models were adjusted for age (as the time scale), sex, education attainment, project, baseline cigarette smoking, alcohol consumption, physical activity (walking/bicycling and exercise), adherence to the mDASH diet, body mass index, systolic blood pressure, and blood levels of fasting glucose, low-density lipoprotein cholesterol, and high-density lipoprotein cholesterol.

Supplementary Material

Supplementary Table 7. Associations between replicated proteins and incident myocardial infarction in the **UK Biobank cohort** in fully adjusted analyses, n=51,613

	Protein abbreviation	HR	Min 95%	Max 95%	P-value
1	GDF15	1.780	1.620	1.960	0.000
2	ADM	2.240	1.810	2.760	6.71E-14
3	KIM-1 (HAVCR1)	1.380	1.270	1.490	2.22E-15
4	TNFRSF11A	1.370	1.200	1.570	4.07E-06
5	PGF	2.050	1.730	2.430	2.22E-16
6	Gal-9 (LGALS9)	1.880	1.590	2.220	1.20E-13
7	METRNL	n/a	-	-	-
8	AMBP	2.480	1.910	3.230	1.24E-11
9	TNFRSF10A	1.590	1.380	1.840	3.18E-10
10	OPG (TNFRSF11B)	1.970	1.660	2.340	1.04E-14
11	PRSS8	1.510	1.330	1.720	5.61E-10
12	CHI3L1	1.260	1.190	1.350	5.04E-13
13	IGFBP-1	1.080	1.030	1.130	0.001167
14	CTSL1 (CTSL)	1.540	1.240	1.910	0.000109
15	VSIG2	1.260	1.150	1.380	1.78E-06
16	U-PAR (PLAUR)	2.250	1.880	2.690	0
17	RARRES2	1.350	1.210	1.500	6.43E-08
18	TNF-R1 (TNFRSF1A)	1.840	1.560	2.180	7.61E-13
19	IL6	1.210	1.150	1.290	4.56E-11
20	TFPI	1.450	1.230	1.710	8.02E-06
21	FS (FST)	1.210	1.080	1.350	0.000835
22	Gal-4 (LGALS4)	1.390	1.260	1.530	3.48E-11
23	FGF-23	1.210	1.100	1.320	3.71E-05
24	MMP-3	1.370	1.230	1.520	5.30E-09

25	REN	1.240	1.160	1.320	2.77E-10
26	CTSZ	1.490	1.260	1.770	4.12E-06
27	CTSD	1.300	1.150	1.470	3.34E-05
28	LILRA5	1.730	1.470	2.050	1.11E-10
29	CLEC5A	1.550	1.300	1.840	6.58E-07
30	TNFRSF14	1.640	1.380	1.940	8.56E-09
31	FAM3C	1.650	1.380	1.960	2.38E-08
32	NPDC1	1.580	1.360	1.830	1.03E-09
33	OPN (SPP1)	1.420	1.260	1.590	4.37E-09
34	CCL15	1.350	1.210	1.520	2.47E-07
35	CXCL16	2.100	1.680	2.620	5.48E-11
36	MMP-12	1.700	1.550	1.860	0
37	TR (TFRC)	1.190	1.030	1.360	0.01749
38	REG4	1.400	1.260	1.550	3.30E-10
39	SELP	1.210	1.100	1.330	9.13E-05
40	TFF3	1.420	1.280	1.570	1.11E-11
41	TNF-R2 (TNFRSF1B)	1.490	1.340	1.650	1.94E-13
42	TRAIL-R2 (TNFRSF10B)	1.340	1.240	1.440	5.55E-15
43	TIMP4	1.390	1.220	1.590	7.62E-07
44	TR-AP (ACP5)	1.380	1.170	1.620	0.000138
45	CD1C	0.680	0.550	0.850	0.000458

Hazard ratios (HR) with 95% confidence intervals (CI) are expressed per standard deviation unit change in protein measurements. In UK Biobank, models were adjusted for age, ethnicity (White, Black, Asian, and other), Townsend social deprivation index, smoking (never, previous or current), prevalent diabetes (yes/No), systolic blood pressure, body mass index, low-density lipoprotein cholesterol, and high-density lipoprotein cholesterol, and fasting time.

METRNL was not available in the UK Biobank data and, therefore, was not replicated.

Supplementary Material

Supplementary Table 8. Associations between circulating proteins and incident myocardial infarction in **women** in the SIMPLER cohort and the UK Biobank cohort

	**Protein abbreviation**	**HR**	**Min 95%**	**Max 95%**	**P-value**
**SIMPLER** (n = 7,423)					
1	GDF15	1.679	1.444	1.952	**1.60E-11**
2	MMP-3	1.301	1.123	1.509	**4.80E-04**
3	CXCL16	1.301	1.127	1.501	**3.10E-04**
4	Gal-4	1.298	1.118	1.507	**6.00E-04**
5	TNFRSF14	1.295	1.120	1.498	**5.00E-04**
6	EPHB4	1.289	1.083	1.534	**4.30E-03**
7	FGF-23	1.269	1.122	1.435	**1.40E-04**
8	U-PAR	1.249	1.114	1.400	**1.40E-04**
9	CCL15	1.229	1.077	1.403	**2.20E-03**
10	REG4	1.224	1.070	1.401	**3.30E-03**
11	IL-1RT1	1.220	1.050	1.417	**9.40E-03**
12	MMP-2	1.220	1.052	1.415	**8.70E-03**
13	IGFBP-7	1.214	1.065	1.383	**3.70E-03**
**UK Biobank** (n = 28,313)					
1	GDF15	1.880	1.630	2.180	**0.000**
2	MMP-3	1.530	1.290	1.810	**7.69E-07**
3	CXCL16	2.870	1.990	4.150	**1.75E-08**
4	Gal-4	1.580	1.340	1.860	**4.53E-08**
5	TNFRSF14	2.090	1.580	2.770	**3.03E-07**
6	EPHB4	2.270	1.650	3.130	**5.41E-07**
7	FGF-23	1.370	1.210	1.560	**1.07E-06**
8	U-PAR	2.990	2.200	4.070	**3.15E-12**
9	CCL15	1.610	1.330	1.940	**7.65E-07**
10	REG4	1.500	1.250	1.790	**8.68E-06**
11	IL-1RT1	1.730	1.160	2.580	**0.007434**
12	MMP-2	n/a	-	-	-
13	IGFBP7	1.810	1.390	2.360	**1.25E-05**

Supplementary Material

Hazard ratios (HR) with 95% confidence intervals (CI) are expressed per standard deviation unit change in protein measurements. In SIMPLER, models were adjusted for age (as the time scale), education attainment, project, baseline cigarette smoking, alcohol consumption, physical activity (walking/bicycling and exercise**)**, adherence to the mDASH diet, body mass index, systolic blood pressure, and blood levels of fasting glucose, low-density lipoprotein cholesterol, and high-density lipoprotein cholesterol. In UK Biobank, models were adjusted for age, ethnicity (White, Black, Asian, and other), Townsend social deprivation index, smoking (never, previous or current), prevalent diabetes (yes/No), systolic blood pressure, body mass index, low-density lipoprotein cholesterol, and high-density lipoprotein cholesterol, and fasting time.

MMP-2 was not available in the UK Biobank data and, therefore, was not replicated.
P-values < 0.05 are highlighted in bold.

Supplementary Material

Supplementary Table 9. Associations between circulating proteins and incident myocardial infarction in **men** in the SIMPLER cohort and the UK Biobank cohort

	Protein abbreviation	HR	Min 95%	Max 95%	P-value
**SIMPLER** (n = 4,328)					
1	GDF15	1.230	1.042	1.452	**1.50E-02**
2	MMP-3	1.064	0.888	1.276	5.00E-01
3	CXCL16	1.068	0.927	1.230	3.60E-01
4	Gal-4	1.124	0.969	1.304	1.20E-01
5	TNFRSF14	1.133	0.981	1.309	9.00E-02
6	EPHB4	1.091	0.951	1.251	2.20E-01
7	FGF-23	1.132	0.992	1.293	6.60E-02
8	U-PAR	1.144	0.984	1.329	7.90E-02
9	CCL15	1.118	0.970	1.289	1.20E-01
10	REG4	1.105	0.957	1.276	1.70E-01
11	IL-1RT1	1.023	0.884	1.185	7.60E-01
12	MMP-2	0.990	0.854	1.148	9.00E-01
13	IGFBP-7	0.975	0.840	1.131	7.40E-01
**UK Biobank** (n = 23,300)					
1	GDF15	1.730	1.520	1.960	**2.22E-16**
2	MMP-3	1.290	1.130	1.470	**0.000209**
3	CXCL16	1.740	1.310	2.290	**0.000106**
4	Gal-4	1.290	1.150	1.460	**3.03E-05**
5	TNFRSF14	1.430	1.160	1.760	**0.000854**
6	EPHB4	1.290	1.000	1.670	0.050529
7	FGF-23	1.090	0.960	1.230	0.205838
8	U-PAR	1.950	1.560	2.430	**3.41E-09**
9	CCL15	1.220	1.050	1.410	**0.007732**
10	REG4	1.340	1.170	1.520	**1.12E-05**
11	IL-1RT1	1.190	0.890	1.590	0.231623
12	MMP-2	n/a	-	-	-
13	IGFBP-7	1.400	1.150	1.700	**0.000611**

Supplementary Material

Hazard ratios (HR) with 95% confidence intervals (CI) are expressed per standard deviation unit change in protein measurements. In SIMPLER, models were adjusted for age (as the time scale), education attainment, project, baseline cigarette smoking, alcohol consumption, physical activity (walking/bicycling and exercise**)**, adherence to the mDASH diet, body mass index, systolic blood pressure, and blood levels of fasting glucose, low-density lipoprotein cholesterol, and high-density lipoprotein cholesterol. In UK Biobank, models were adjusted for age, ethnicity (White, Black, Asian, and other), Townsend social deprivation index, smoking (never, previous or current), prevalent diabetes (yes/No), systolic blood pressure, body mass index, low-density lipoprotein cholesterol, and high-density lipoprotein cholesterol, and fasting time.

MMP-2 was not available in the UK Biobank data and, therefore, was not replicated.
P-values < 0.05 are highlighted in bold.

Supplementary Material

Supplementary Table 10. Results of Mendelian randomization analysis for identified protein-related SNPs and the risk of myocardial infarction

	Protein Abbreviation	Source	Beta	SE	P-value
1	GDF15	Consortium	-0.043	0.046	0.353
2	ADM	Consortium	-0.089	0.068	0.191
		FinnGen	0.097	0.067	0.147
		Combined	0.005	0.048	0.908
3	KIM-1 (HAVCR1)	Consortium	-0.002	0.024	0.934
		FinnGen	0.023	0.024	0.327
		Combined	0.011	0.017	0.524
4	TNFRSF11A*		-	-	-
5	PGF	Consortium	-0.180	0.068	**0.008**
		FinnGen	-0.087	0.067	0.195
		Combined	-0.133	0.048	**0.005**
6	Gal-9 (LGALS9)	FinnGen	-0.064	0.158	0.688
7	METRNL*		-	-	-
8	AMBP*		-	-	-
9	TNFRSF10A	Consortium	0.050	0.035	0.155
		FinnGen	-0.015	0.034	0.653
		Combined	0.016	0.025	0.503
10	OPG (TNFRSF11B)	Consortium	-0.033	0.133	0.807
		FinnGen	-0.211	0.133	0.113
		Combined	-0.122	0.094	0.196
11	PRSS8	Consortium	0.012	0.100	0.908
		FinnGen	-0.074	0.095	0.436
		Combined	-0.033	0.069	0.627
12	CHI3L1	Consortium	-0.043	0.037	0.253
		FinnGen	-0.038	0.036	0.291
		Combined	-0.040	0.026	0.120
13	IGFBP-1	Consortium	0.017	0.234	0.943
		FinnGen	-0.332	0.230	0.150

		Combined	-0.160	0.164	0.329
14	CTSL1 (CTSL)*		-	-	-
15	VSIG2	Consortium	-0.008	0.057	0.892
		FinnGen	-0.106	0.053	**0.048**
		Combined	-0.060	0.039	0.123
16	U-PAR (PLAUR)	Consortium	-0.044	0.083	0.600
		FinnGen	0.068	0.092	0.460
		Combined	0.007	0.062	0.915
17	RARRES2	Consortium	0.029	0.054	0.597
		FinnGen	0.121	0.053	**0.023**
		Combined	0.076	0.038	**0.045**
18	TNF-R1 (TNFRSF1A)	FinnGen	-0.327	0.157	**0.037**
19	IL6*		-	-	-
20	TFPI	Consortium	-0.092	0.037	**0.012**
		FinnGen	-0.060	0.036	0.096
		Combined	-0.076	0.026	**0.003**
21	FS (FST)	Consortium	0.115	0.075	0.129
		FinnGen	0.262	0.073	**0.000**
		Combined	0.190	0.053	**0.000**
22	Gal-4 (LGALS4)	Consortium	0.156	0.148	0.293
		FinnGen	0.115	0.119	0.336
		Combined	0.131	0.093	0.159
23	FGF-23	Consortium	0.008	0.100	0.940
		FinnGen	0.042	0.099	0.670
		Combined	0.025	0.070	0.723
24	MMP-3	Consortium	0.078	0.050	0.117
		FinnGen	0.038	0.043	0.387
		Combined	0.055	0.033	0.093
25	REN	Consortium	0.230	0.146	0.115
		FinnGen	0.291	0.146	**0.046**

		Combined	0.261	0.103	**0.012**
26	CTSZ	Consortium	0.044	0.034	0.197
		FinnGen	0.012	0.036	0.727
		Combined	0.029	0.025	0.240
27	CTSD	Consortium	-0.028	0.039	0.486
		FinnGen	-0.042	0.045	0.354
		Combined	-0.034	0.030	0.256
28	LILRA5	Consortium	-0.073	0.040	0.065
		FinnGen	-0.027	0.035	0.446
		Combined	-0.047	0.026	0.074
29	CLEC5A	Consortium	0.051	0.048	0.287
		FinnGen	0.022	0.048	0.651
		Combined	0.036	0.034	0.284
30	TNFRSF14	Consortium	0.122	0.092	0.186
		FinnGen	0.095	0.089	0.288
		Combined	0.108	0.064	0.093
31	FAM3C	Consortium	-0.020	0.100	0.843
		FinnGen	-0.207	0.104	**0.047**
		Combined	-0.110	0.072	0.129
32	NPDC1*		-	-	-
33	OPN (SPP1)	Consortium	-0.130	0.122	0.289
		FinnGen	-0.058	0.115	0.612
		Combined	-0.092	0.084	0.274
34	CCL15	Consortium	-0.010	0.460	0.983
		FinnGen	0.111	0.296	0.708
		Combined	0.075	0.249	0.762
35	CXCL16	Consortium	-0.041	0.059	0.490
		FinnGen	-0.034	0.054	0.528
		Combined	-0.037	0.040	0.351
36	MMP-12	Consortium	-0.042	0.043	0.327
		FinnGen	0.003	0.041	0.944

		Combined	-0.018	0.029	0.532
37	TR (TFRC)	Consortium	0.004	0.037	0.916
		FinnGen	-0.007	0.041	0.861
		Combined	-0.001	0.027	0.971
38	REG4	FinnGen	0.017	0.055	0.755
39	SELP	Consortium	0.074	0.053	0.163
		FinnGen	-0.069	0.039	0.076
		Combined	-0.019	0.031	0.544
40	TFF3*		-	-	-
41	TNF-R2 (TNFRSF1B)	Consortium	-0.061	0.084	0.469
		FinnGen	-0.225	0.088	**0.010**
		Combined	-0.139	0.061	**0.021**
42	TRAIL-R2 (TNFRSF10B)	Consortium	0.009	0.036	0.808
		FinnGen	0.010	0.034	0.760
		Combined	0.010	0.025	0.698
43	TIMP4	Consortium	0.033	0.038	0.386
44	TR-AP (ACP5)	Consortium	-0.004	0.053	0.934
		FinnGen	-0.001	0.054	0.992
		Combined	-0.002	0.038	0.947
45	CD1C	Consortium	-0.100	0.183	0.586
		FinnGen	-0.202	0.154	0.191
		Combined	-0.160	0.118	0.177

SE, standard error. *TNFRSF11A, METRNL, AMBP, CTSL1 (CTSL), IL6, NPDC1, and TFF3 were not available in the outcome source or had no suitable instrumental variable in protein-related GWAS data or proxy SNPs.

Supplementary Material

Supplementary Table 11. Associations between 45 proteins identified in the primary analysis and overall mortality in SIMPLER in fully adjusted analyses (n = 12,314)

	Protein abbreviation	HR	Min 95%	Max 95%	P-value*
1	GDF15	1.517	1.446	1.592	3.90E-65
2	ADM	1.349	1.274	1.428	1.10E-24
3	KIM1	1.332	1.273	1.392	4.80E-36
4	TNFRSF10A	1.326	1.264	1.391	5.60E-31
5	U-PAR	1.287	1.238	1.338	3.60E-37
6	TNF-R1	1.247	1.193	1.304	1.80E-22
7	OPN	1.245	1.195	1.298	1.30E-25
8	IL6	1.245	1.199	1.293	2.60E-30
9	PGF	1.235	1.174	1.299	3.90E-16
10	OPG	1.230	1.178	1.285	8.80E-21
11	CHI3L1	1.225	1.173	1.280	1.10E-19
12	TFF3	1.225	1.181	1.271	1.30E-27
13	CTSL1	1.225	1.167	1.286	2.60E-16
14	TNFRSF11A	1.225	1.171	1.282	1.10E-18
15	MMP-12	1.217	1.162	1.275	9.70E-17
16	FGF-23	1.215	1.173	1.258	9.70E-28
17	TRAIL-R2	1.212	1.171	1.254	1.60E-28
18	IGFBP-1	1.210	1.150	1.273	2.70E-13
19	TNFRSF14	1.206	1.156	1.259	5.50E-18
20	TIMP4	1.204	1.156	1.253	2.70E-19
21	CCL15	1.201	1.152	1.253	1.10E-17
22	CLEC5A	1.200	1.150	1.253	4.50E-17
23	TNF-R2	1.198	1.152	1.245	3.70E-20
24	Gal-9	1.193	1.128	1.261	5.30E-10
25	NPDC1	1.191	1.141	1.244	1.60E-15
26	FS	1.190	1.138	1.244	1.80E-14
27	TR	1.188	1.140	1.238	2.30E-16
28	REG4	1.187	1.139	1.237	5.00E-16
29	Gal-4	1.187	1.134	1.242	1.60E-13
30	PRSS8	1.186	1.117	1.258	2.20E-08
31	VSIG2	1.183	1.132	1.238	1.80E-13

32	METRNL	1.181	1.122	1.243	1.90E-10
33	LILRA5	1.175	1.119	1.235	1.10E-10
34	AMBP	1.159	1.095	1.226	3.00E-07
35	MMP-3	1.150	1.095	1.208	3.00E-08
36	FAM3C	1.142	1.091	1.195	1.40E-08
37	REN	1.140	1.086	1.197	1.40E-07
38	RARRES2	1.131	1.080	1.185	2.20E-07
39	CTSZ	1.129	1.077	1.184	6.10E-07
40	TFPI	1.119	1.068	1.173	2.80E-06
41	CXCL16	1.116	1.071	1.164	2.70E-07
42	CTSD	1.110	1.064	1.158	1.40E-06
43	SELP	1.104	1.059	1.150	3.10E-06
44	TR-AP	1.065	1.023	1.108	2.00E-03
45	CD1C	0.928	0.890	0.968	5.40E-04

Hazard ratios (HR) with 95% confidence intervals (CI) are expressed per standard deviation unit change in protein measurements. The models were adjusted for age (as the time scale), sex, education attainment, project, baseline cigarette smoking, alcohol consumption, physical activity (walking/bicycling and exercise), adherence to the mDASH diet, body mass index, systolic blood pressure, and blood levels of fasting glucose, low-density lipoprotein cholesterol, and high-density lipoprotein cholesterol.
P-values are <0.05, all passed FDR.

Supplementary Material

Supplementary Table 12. Druggability of proteins associated with MI in Mendelian randomization analysis

**Protein Abbreviation** (alternative abbreviation) and **Drugbank ID***	**UniProt ID**	**Drug or component name***	**Indication or biological function***	**Drug group***	**Detailed information (reference)***
**REN**	P00797				
DB00212		Remikiren	High specificity renin inhibitor	experimental	Details
DB09026		Aliskiren	Renin inhibitor used to manage hypertension	Approved by FDA, investigational	Details
DB01844		N,N-dimethylformamide	N/A	experimental	Details
DB02296		Isoamyl alcohol	N/A	experimental	Details
DB02803		3-Phenyl-1,2-Propandiol	N/A	experimental	Details
DB03395		Enalkiren	N/A	experimental	Details
Details about other experimental drugs and clinical candidates (total n=21) are available: https://go.drugbank.com/bio_entities/BE0000270 https://www.ebi.ac.uk/chembl/target_report_card/CHEMBL286/					
**FS (FST)**	P19883				
DB01666		D-Myo-Inositol-Hexasulphate	N/A	experimental	Details
**RARRES2**	Q99969	N/A			
**TFPI**	P10646				
DB00036		Coagulation factor VIIa Recombinant Human	A form of recombinant human coagulation Factor VII used to treat hemophilia A and B.	approved	Details

DB14562		Andexanet alfa	A recombinant human coagulation Factor Xa that promotes blood coagulation. It is indicated for patients treated with rivaroxaban and apixaban, when reversal of anticoagulation is needed	approved, investigational	Details
**TNF-R2 (TNFRSF1B)**	P20333				
DB11626		Tasonermin	Used as adjunct in combination to treat irresectable soft tissue sarcoma of the limb	approved	Details
**PGF**	P49763				
DB08885		Aflibercept	Used for the treatment of neovascular (Wet) age-related macular degeneration (AMD), macular edema following retinal vein occlusion (RVO), diabetic macular edema (DME), diabetic retinopathy (DR), and retinopathy of prematurity (ROP).	approved	Details
**TNF-R1 (TNFRSF1A)**	P19438				
DB03507		6-[3-(4-Morpholinyl)Propyl]-2-(3-Nitrophenyl)-5-Thioxo-5,6,-Dihydro-7h-Thienol[2',3':4,5]Pyrrolo[1,2-C]Imidazol-7-One	N/A	experimental	Details
DB11626		Tasonermin	A tumor necrosis factor alpha used along with surgery to remove soft tissue sarcomas of the limbs.	approved	Details
		GSK-1995057	Respiratory Tract Diseases	Phase 1 #	

* Information obtained from the DrugBank
# Information on experimental drugs obtained from the ChEMBL

Supplementary Material

Supplementary Figure 1. Flow chart and study design based on SIMPLER and UK Biobank cohorts and Mendelian randomization analysis.

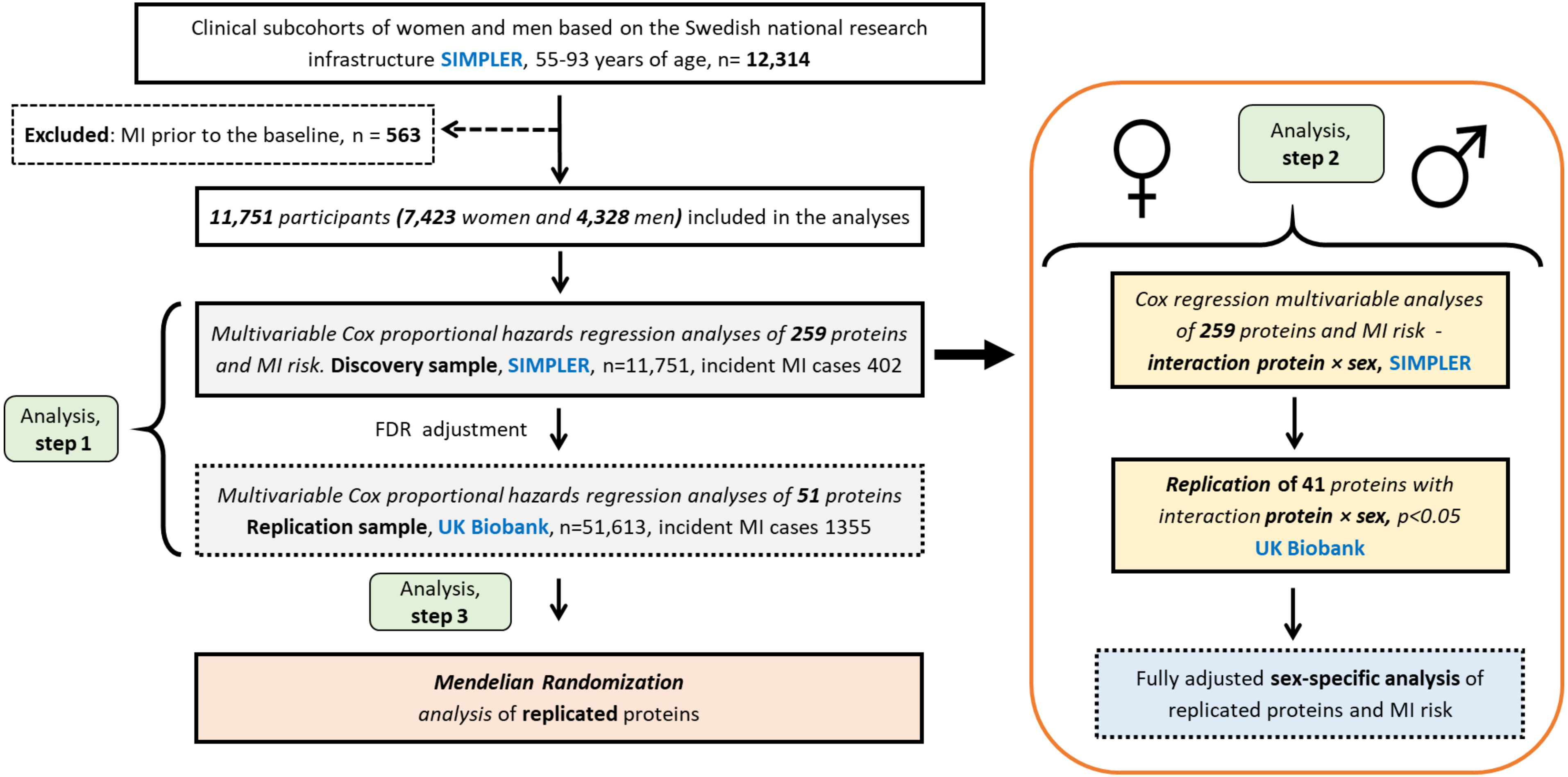

Supplementary Material

Supplementary Figure 2. Correlation between 45 replicated circulating proteins associated with incident myocardial infarction in the analysis based on SIMPLER.
Heat map of Pearson's correlation coefficients. A color-coded label of correlation coefficients is shown on the right side of the heat map.

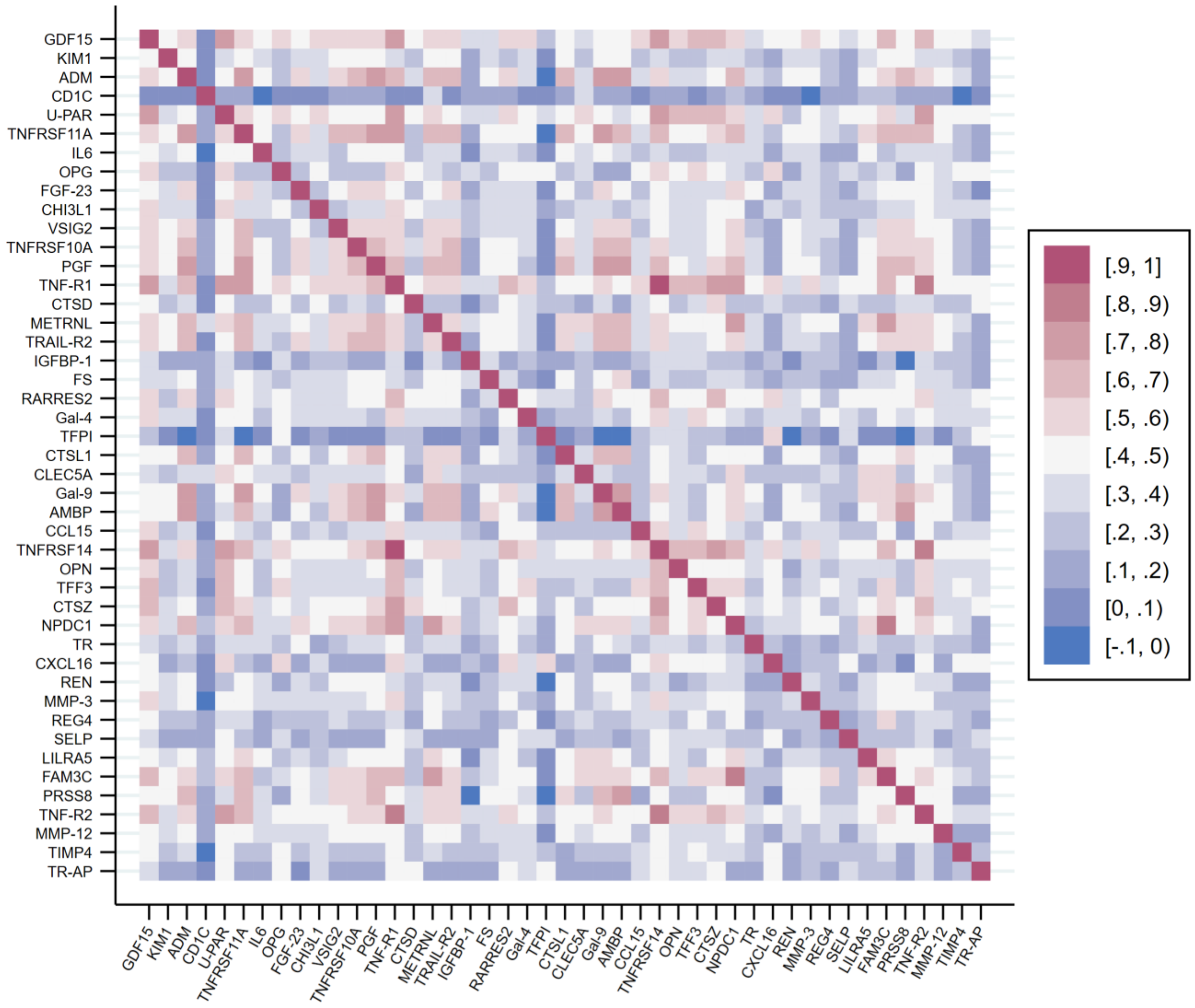

Supplement: ehae658_Supplementary_Data [file ehae658_supplementary_data.pdf]
